# Screening for fetal growth restriction with universal third trimester ultrasonography in nulliparous women in the Pregnancy Outcome Prediction (POP) study: a prospective cohort study

**DOI:** 10.1016/S0140-6736(15)00131-2

**Published:** 2015-11-21

**Authors:** Ulla Sovio, Ian R White, Alison Dacey, Dharmintra Pasupathy, Gordon C S Smith

**Affiliations:** a Department of Obstetrics and Gynaecology, University of Cambridge, National Institute for Health Research Cambridge Comprehensive Biomedical Research Centre, Cambridge, UK; b Medical Research Council Biostatistics Unit, Institute of Public Health, Cambridge, UK; c Division of Women's Health, Women's Health Academic Centre, King's Health Partners, King's College London, Guy's and St Thomas' NHS Foundation Trust, London, UK

## Abstract

**Background:**

Fetal growth restriction is a major determinant of adverse perinatal outcome. Screening procedures for fetal growth restriction need to identify small babies and then differentiate between those that are healthy and those that are pathologically small. We sought to determine the diagnostic effectiveness of universal ultrasonic fetal biometry in the third trimester as a screening test for small-for-gestational-age (SGA) infants, and whether the risk of morbidity associated with being small differed in the presence or absence of ultrasonic markers of fetal growth restriction.

**Methods:**

The Pregnancy Outcome Prediction (POP) study was a prospective cohort study of nulliparous women with a viable singleton pregnancy at the time of the dating ultrasound scan. Women participating had clinically indicated ultrasonography in the third trimester as per routine clinical care and these results were reported as usual (selective ultrasonography). Additionally, all participants had research ultrasonography, including fetal biometry at 28 and 36 weeks' gestational age. These results were not made available to participants or treating clinicians (universal ultrasonography). We regarded SGA as a birthweight of less than the 10th percentile for gestational age and screen positive for SGA an ultrasonographic estimated fetal weight of less than the 10th percentile for gestational age. Markers of fetal growth restriction included biometric ratios, utero-placental Doppler, and fetal growth velocity. We assessed outcomes for consenting participants who attended research scans and had a livebirth at the Rosie Hospital (Cambridge, UK) after the 28 weeks' research scan.

**Findings:**

Between Jan 14, 2008, and July 31, 2012, 4512 women provided written informed consent of whom 3977 (88%) were eligible for analysis. Sensitivity for detection of SGA infants was 20% (95% CI 15–24; 69 of 352 fetuses) for selective ultrasonography and 57% (51–62; 199 of 352 fetuses) for universal ultrasonography (relative sensitivity 2·9, 95% CI 2·4–3·5, p<0·0001). Of the 3977 fetuses, 562 (14·1%) were identified by universal ultrasonography with an estimated fetal weight of less than the 10th percentile and were at an increased risk of neonatal morbidity (relative risk [RR] 1·60, 95% CI 1·22–2·09, p=0·0012). However, estimated fetal weight of less than the 10th percentile was only associated with the risk of neonatal morbidity (p_interaction_=0·005) if the fetal abdominal circumference growth velocity was in the lowest decile (RR 3·9, 95% CI 1·9–8·1, p=0·0001). 172 (4%) of 3977 pregnancies had both an estimated fetal weight of less than the 10th percentile and abdominal circumference growth velocity in the lowest decile, and had a relative risk of delivering an SGA infant with neonatal morbidity of 17·6 (9·2–34·0, p<0·0001).

**Interpretation:**

Screening of nulliparous women with universal third trimester fetal biometry roughly tripled detection of SGA infants. Combined analysis of fetal biometry and fetal growth velocity identified a subset of SGA fetuses that were at increased risk of neonatal morbidity.

**Funding:**

National Institute for Health Research, Medical Research Council, Sands, and GE Healthcare.

## Introduction

Use of ultrasonography to identify small-for-gestational-age (SGA) infants is widespread in contemporary obstetric practice.^1^, ^2^ In the USA, UK, and many other countries women are not routinely scanned in late pregnancy, but are selected for third trimester ultrasonography on the basis of pre-pregnancy risk factors, development of obstetric complications, and serial measurement of symphyseal-fundal height.^2^, ^3^ This approach identifies a third of SGA infants or fewer,^4^, ^5^, ^6^ and unidentified SGA is a common finding in perinatal deaths.^7^, ^8^ However, a meta-analysis^9^ of nine trials assessing universal late pregnancy ultrasonography, including about 27 000 women, showed no beneficial effect, which led to the recommendation that it should not be offered routinely in the third trimester.^2^, ^3^

Assessment of a screening programme could yield a negative result for three major reasons. First, the screening test could perform poorly—ie, have poor diagnostic effectiveness. Second, screening might not be coupled with use of an effective intervention—ie, the screening programme would not be clinically effective. Third, both the screening test and intervention could be effective, but the studies analysed might be methodologically flawed—eg, they might be underpowered.^10^ A screening study can be designed only if the diagnostic effectiveness of the screening test has been well characterised. The National Institute for Health and Care Excellence (NICE) in the UK did a thorough systematic review of the evidence about the diagnostic effectiveness of universal screening for SGA using ultrasonography for their 2008 Antenatal Care guideline.^3^ They concluded that, “the methods by which [SGA] can be identified antenatally are poorly developed or not tested by rigorous methodology”. Furthermore, SGA is frequently used as a proxy for fetal growth restriction (FGR). However, in reality many SGA infants are physiologically small. Very little information is available about the ability of universal ultrasonography to identify those SGA fetuses that are at increased risk of morbidity (panel).

The aims of this study were to compare the diagnostic effectiveness of universal ultrasound as a screening test for SGA compared with selective ultrasound and to establish which, if any, of a series of previously described ultrasonic markers of FGR identified those SGA fetuses at an increased risk of an adverse outcome.

## Methods

### Study design and participants

In this prospective cohort Pregnancy Outcome Prediction (POP) study, nulliparous women attending for their dating ultrasound scan at the Rosie Hospital (Cambridge, UK) between Jan 14, 2008, and July 31, 2012, with a viable pregnancy were eligible to participate. The protocol has been published elsewhere.^11^ The only clinical exclusion criterion was multiple pregnancy. Women were selected for clinically indicated ultrasound scans in the third trimester as per routine clinical care, and the results of these scans were reported (selective ultrasonography). All women in the cohort also had research ultrasound scans in which both the women and the clinicians caring for them were masked to the results (universal ultrasonography). After delivery, the results of the research scans were unmasked and their associations with birthweight less than the 10th percentile and neonatal morbidity were assessed. This study was designed to generate level 1 evidence of diagnostic effectiveness, as defined by the latest NICE Guideline at the time.^12^ Reporting of this study conforms to the STROBE (The Strengthening the Reporting of Observational Studies in Epidemiology) statement.

Ethical approval for the study was obtained from the Cambridgeshire 2 Research Ethics Committee (reference 07/H0308/163) and approval to study data routinely gathered from non-participants was obtained from the South Central (Berkshire) Research Ethics Committee (reference 12/SC/0344). Participants provided written informed consent.

### Procedures

Women who agreed to participate were given follow-up appointments at about 20, 28, and 36 weeks' gestation in the National Institute for Health Research Cambridge Clinical Research Facility (Cambridge, UK). All research scans after the dating scan were done with a Voluson *i* system (GE Healthcare, Fairfield CT, USA) by one of a team of six sonographers, all of whom received standard training. All ultrasound examinations followed the same protocols as those used in the clinical service.^13^, ^14^ At the 20 week research appointment, participants were given a novel questionnaire we created to obtain details about their medical history and demographic characteristics.^11^ The 20 week scan had both routine (review of fetal anatomy and biometric measurements) and research (uterine and umbilical artery Doppler flow velocimetry) elements. Women were informed about routine elements (any concerns about the fetal anatomy and of the fetal measurements at the 20 week scan), but women and clinicians were masked to the research elements (results of the uterine and umbilical Dopplers). At the 28 and 36 week research appointments, umbilical and uterine artery Doppler flow velocimetry were repeated, and ultrasonographic measurement of fetal biparietal diameter, head circumference, abdominal circumference, and femur length were also done using standard techniques. An estimated fetal weight (EFW) percentile was calculated by use of the Hadlock equations and reference standard.^15^, ^16^ Uteroplacental Dopplers, biometry, and EFW results from the research ultrasound scans at 28 and 36 weeks were not reported to the participant or the clinician. However, both were informed about incidental findings, specifically previously undiagnosed placenta praevia, severe oligohydramnios (amniotic fluid index <5), a previously undiagnosed fetal abnormality, or non-cephalic presentation at the time of the 36 week scan.

Gestational age was defined on the basis of ultrasonographic estimation at the time of the first scan, as recommended.^3^ Distributions of all measurements in the research scans were similar to previously reported reference cohorts (appendix). Summary statistics for reproducibility and reliability of research scans (assessed by two sonographers, scanning the same woman twice at the same appointment, each masked to the results of the other's scan) are tabulated for 45 women at 20 weeks' gestation and 44 women at 36 weeks' gestation (appendix). Coefficients of variation were less than 5% for fetal biometry and EFW, and between 5% and 10% for uteroplacental Doppler at both timepoints.

Women were selected for additional, clinically indicated scans in the third trimester of pregnancy as per routine clinical care, using local and national guidelines (eg, the NICE Guidelines on low risk women,^3^ women with diabetes,^17^ and women with hypertensive disorders^18^). Women were also screened with serial measurement of the symphyseal-fundal height. All women carried their maternity notes, which included a chart of the normal range of measurements for fetuses in relation to gestational age. Referral for an ultrasound scan was made by the midwife or doctor providing clinical care. Results of all clinically indicated scans were reported and paper copies were filed in both the participant's hand-held notes and hospital case records.

Screening status in relation to EFW was classified on the basis of the last scan before birth (which could be the 28 week scan or the 36 week scan for universal ultrasonography, depending on the gestational age at delivery). Screen positive was defined as an EFW less than the 10th percentile, using an externally derived reference range^15^, ^16^ (for both selective and universal ultrasonography). Screen negative was defined as an EFW of the 10th percentile or more (both selective and universal ultrasonography), or if no clinically indicated scan had been done at gestational age of 26 weeks or later (only selective ultrasonography).

### Outcomes

Inclusion criteria for analysis were that women attended research scans booked before delivery and had a live birth at the Rosie Hospital. We excluded women who delivered before their 28 week scan appointment from the analysis. The results of clinically indicated scans and the outcome of the pregnancy were ascertained by individual review of all paper-case records by research midwives, and by linkage of the research data to the hospitals' electronic databases of ultrasonography (Astraia; Munich, Germany), delivery (Protos; iSoft, Banbury, UK), biochemical tests (Meditech; Westwood, MA, USA), and neonatal intensive care (Badgernet, Clevermed, Edinburgh, UK). The gold standard for SGA was birthweight of less than the 10th percentile for sex and gestational age, calculated from a UK reference.^19^ We also studied severe SGA (birthweight <3rd percentile) as a secondary outcome.

We defined neonatal morbidity as one or more of the following criteria: a 5 min Apgar score of less than 7, delivery with metabolic acidosis (defined as a cord blood pH <7·1 and base deficit >10 mmol/L), or admission to the neonatal unit at term (defined as admission <48 h after birth at ≥37 weeks' gestational age and discharge ≥48 h after admission). We defined severe adverse perinatal outcome as stillbirth or term livebirth associated with neonatal death, hypoxic ischaemic encephalopathy, use of inotropes, need for mechanical ventilation, or severe metabolic acidosis (defined as a cord blood pH <7·0 and base deficit >12 mmol/L: the criteria by which international guidelines define fetal metabolic acidosis, which can be regarded as cause for cerebral palsy during childhood^20^). This group included seven stillbirths, which had been excluded from the main study cohort.

We calculated customised percentiles of EFW on the basis of reported methods,^21^ but used coefficients from the latest model of Gestation-Related Optimal Weight (GROW; version 6.7.3_13 [UK]). We compared associations between population-based EFW and customised EFW of less than the 10th percentile and neonatal morbidity. We analysed other indicators of growth restriction through comparison of the association between an EFW of less than the 10th percentile and neonatal morbidity, in the presence or absence of the given factor. We quantified Doppler flow velocimetry with the pulsatility index,^14^ and uterine artery pulsatility index as the mean pulsatility index of the left and right uterine arteries, classified by the measurement at the 20 week scan.^14^ We classified umbilical artery pulsatility index, head circumference-to-abdominal circumference ratio, and abdominal circumference-to-femur length ratio by the last measurement taken before birth. We quantified all measurements as gestational age adjusted *Z* scores, to account for variation in the exact timing of ultrasound scans (appendix). We quantified growth velocity as the difference in abdominal circumference *Z* score, comparing the last scan before birth and the scan at 20 weeks. For all five of these indices, we generated deciles by use of the distribution in the study cohort. We defined as abnormal the highest deciles of head circumference-to-abdominal circumference ratio, uterine Doppler, and umbilical Doppler in addition to the lowest deciles of abdominal circumference-to-femur length ratio and abdominal circumference growth velocity. We did not investigate other growth indices to reduce the possibility of chance findings due to repeated hypothesis tests. Our study did not include Doppler assessment of blood flow in fetal vessels (eg, ductus venosus or middle cerebral artery).

### Statistical analysis

We used an open-ended recruitment approach, to provide increased power for the less common adverse outcomes with greater cohort size. In our protocol paper^11^ we identified a sample size of 4000 women as providing reasonably precise estimates of sensitivity for outcomes affecting 3% of the population.

We compared continuous variables with a two-sample Wilcoxon rank-sum test and categorical variables with the Pearson χ^2^ test, with a trend test if appropriate, or Fisher's exact test if numbers were small. We compared sensitivity, specificity, false positive rate, and false negative rate using McNemar's test; positive and negative predictive values using weighted generalised score tests;^22^ and likelihood ratios using regression model-based tests.^23^ We did a series of post-hoc sensitivity analyses in which we included women who had defaulted from their research scans (at 28 or 36 weeks), and excluded women who had their research scan results shown to them for any reason, and in which we combined the results of universal and selective ultrasonography. We tested interactions between EFW and ultrasonic markers of FGR in their associations with neonatal morbidity by the Mantel-Haenszel test. We defined significance as p<0·05 (two-sided). We did not make formal adjustments for multiple comparisons and did not adjust for maternal baseline characteristics.

Analyses were done with Stata software (version 13.1) and R software (version 3.0.2).

### Role of the funding source

The funders of this study had no role in study design, data analysis, data interpretation, or writing of the report. The corresponding author and a coauthor (US) had full access to data used in the study. The corresponding author had final responsibility for the decision to submit the paper for publication.

## Results

**Figure 1:**
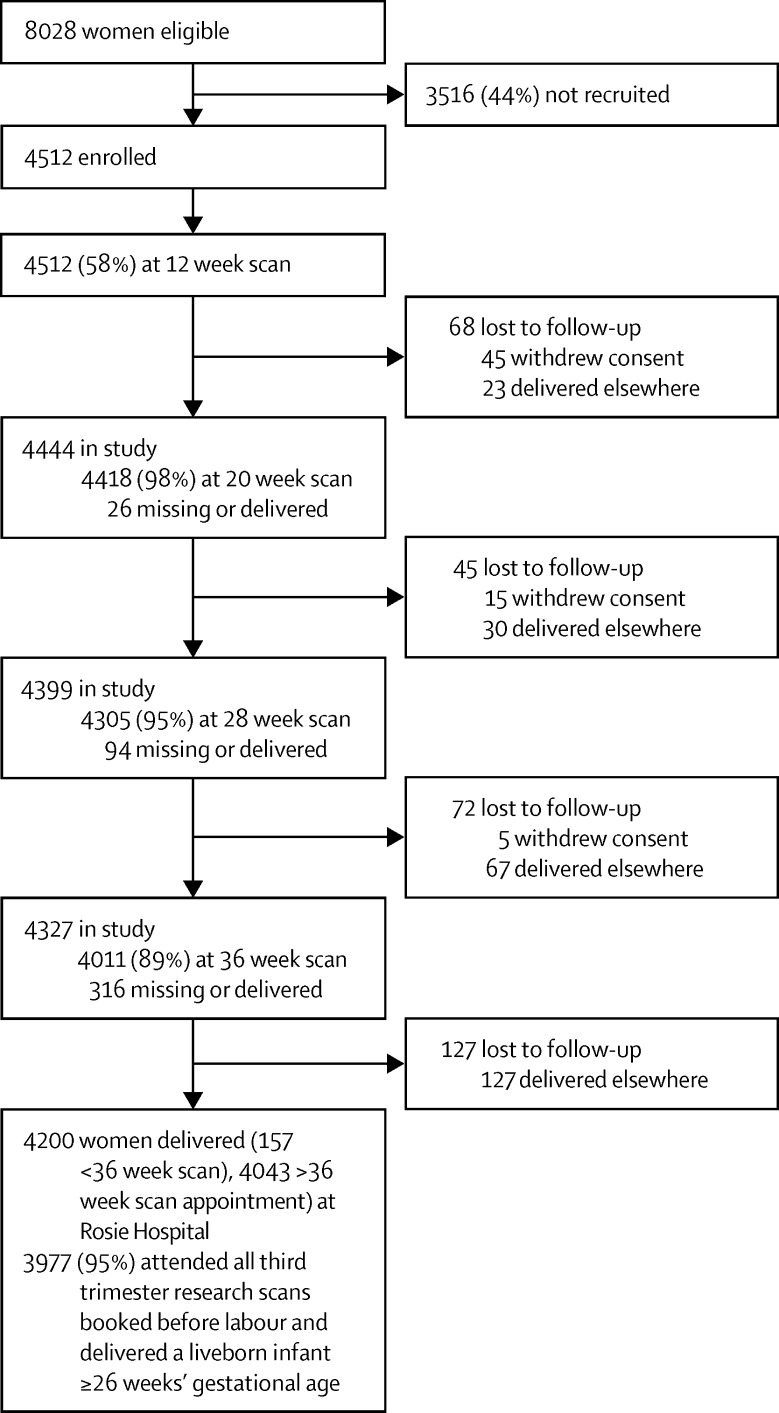
Study profile

Between Jan 14, 2008, and July 31, 2012, we identified 8028 eligible women of whom 4512 (56%) provided written informed consent and were enrolled, whereas 3516 (44%) women declined to participate or were not approached by a study recruiter. Although recruited and non-recruited women were broadly comparable with each other, those recruited were slightly older in age, more often of white ethnic origin, less likely to smoke, more likely to have a caesarean delivery, and had infants of slightly heavier birthweights (appendix). 3977 (88%) of 4512 women recruited were included in the data analysis (figure 1).

1666 (42%) women had a clinically indicated scan including biometry at gestation of 26 weeks or more, and 2311 (58%) women did not (table 1). Women having clinically indicated scans were more likely to be at extremes of maternal age (<20 years and ≥40 years) than those women not having clinically indicated scans and were more likely to have discontinued education early in life (<19 years), a body-mass index greater than 30 kg/m^2^, had previous miscarriages, and to have pre-existing diabetes or to develop gestational diabetes. Average birthweight of the infants born to this group of women was lower, and they had a greater proportion of SGA infants, preterm births, induced labours, and caesarean deliveries than women who did not have clinically indicated scans.

352 (9%) infants had a birthweight of less than the 10th percentile. The last clinically indicated scan before birth recorded an EFW of less than the 10th percentile in 138 (8%) of 1666 women, with 69 of these women going on to have babies with birthweight less than the 10th percentile, yielding a sensitivity of 20% (69 of 352) infants. The last research ultrasound scan before birth recorded an EFW of less than the 10th percentile in 562 women; 199 of these women had babies with a birthweight less than the 10th percentile, yielding a sensitivity of 57% (199 of 352). Table 2, Table 3 compare universal and selective ultrasonography as a two-by-two table and as showing screening summary statistics, respectively. All analyses were repeated with the outcome of severe SGA. Areas under the receiver operating characteristic curve for universal ultrasonography were 0·87 (95% CI 0·85–0·88) for SGA and 0·91 (0·89–0·94) for severe SGA (appendix). Diagnostic effectiveness of the 28 and 36 week research scans are presented separately, and also described for each in relation to the interval between the scan and the delivery date (appendix). Sensitivity analyses generated very similar results to the main analysis (appendix).

**Figure 2:**
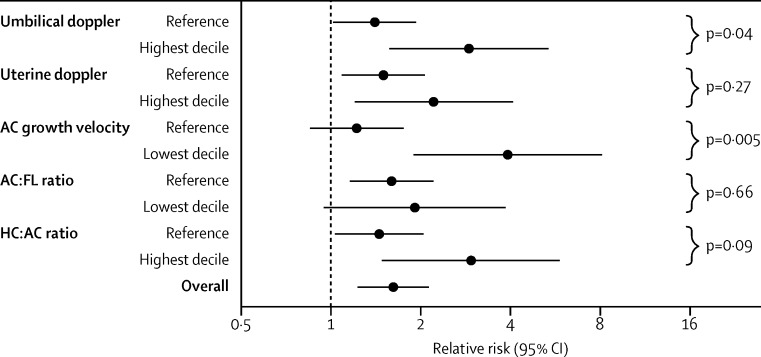
Stratified analyses of the risk of the neonatal composite adverse outcome associated with diagnosis of small-for-gestational-age infants Diagnosis of infants by universal ultrasonography in relation to indicators of fetal growth restriction. The five previously described indices of fetal growth restriction were classified as the extreme decile associated with fetal growth restriction (highest or lowest, as appropriate) compared with the other nine deciles in the cohort. Points are relative risks of neonatal morbidity associated with an ultrasonic diagnosis of a small-for-gestational-age infant at the last scan before birth. p values are a Mantel-Haenszel test calculation of interaction (ie, testing the hypothesis that the association between diagnosis of a small-for-gestation-age infant and neonatal morbidity varies in the two strata). Interactions tested using logistic regression showed almost identical p values. AC=abdominal circumference. FL=femur length. HC=head circumference.

The relative risk of any neonatal morbidity associated with EFW of less than the 10th percentile was 1·6 (95% CI 1·2–2·1, p=0·001; table 4). Definition of EFW with customised percentiles did not result in a stronger association. The association between an EFW lower than the 10th percentile and the risk of neonatal morbidity was then assessed in relation to five previously reported indices of fetal growth restriction (figure 2). Only the measurement of abdominal circumference growth velocity was associated with strong evidence for an interaction (p=0·005). Screen positive fetuses with normal growth velocity were not at increased risk of neonatal morbidity, whereas an EFW of less than the 10th percentile was associated with an increase of 3·9 times (95% CI 1·9–8·1) of neonatal morbidity in infants in the lowest decile of abdominal circumference growth velocity. 172 (4·3%) fetuses had the combination of an ultrasonic diagnosis of SGA plus the lowest decile of abdominal circumference growth velocity from universal ultrasonography. This combination was associated with a relative risk of any morbidity of 2·5 (95% CI 1·7–3·6) and relative risk of delivering an SGA infant with neonatal morbidity of 17·6 (9·2–34·0; table 4). Similar associations were reported when the analysis was repeated for severe adverse perinatal outcome, with a relative risk of 2·9 (95% CI 1·0–8·3, p=0·058) for any severe adverse outcome and 39·8 (95% CI 3·6–436·6, p=0·007) for severe adverse outcome in an SGA infant. We repeated all analyses of abdominal circumference growth velocity using abdominal circumference growth charts generated by the Fetal Growth Longitudinal Study component of the INTERGROWTH-21^st^ Project,^25^ an international consortium that established fetal growth standards using methods recommended by WHO. All associations were very similar when the standards from the INTERGROWTH-21^st^ Project were used (appendix). The combination of ultrasonic diagnosis of SGA infants plus lowest decile of abdominal circumference growth velocity (defined by the INTERGROWTH-21^st^ Project standards) was associated with a relative risk of 2·5 (95% CI 1·7–3·5, p<0·0001) for any morbidity, 17·6 (9·4–33·0, p<0·0001) for delivering an SGA infant with neonatal morbidity, 2·5 (0·9–7·0, p=0·09) for severe adverse perinatal outcome, and 33·4 (3·0–366·6, p=0·009) for delivering an SGA infant with severe adverse perinatal outcome). Finally, no indicator of FGR was associated with adverse outcome if the EFW was above the 10th percentile (appendix).

## Discussion

Fetal growth restriction is associated with many adverse outcomes including stillbirth,^26^ neonatal death,^27^ hypoxic ischaemic encephalopathy,^28^ cerebral palsy,^29^ special educational needs,^30^ and many diseases in adult life.^31^ The present standard of care in the USA,^2^ UK,^3^ and many other countries is that women are selected for third trimester ultrasonographic fetal biometry on the basis of specific indications. From our study of a population of nulliparous women of mixed risk with a singleton pregnancy, we showed that selective use of ultrasonography identified one in five infants with a birthweight of less than the 10th percentile, which is similar to reports from other centres.^4^, ^5^, ^6^ Additionally, a policy of screening with universal ultrasonographic estimation of fetal weight at 28 and 36 weeks' gestational age roughly tripled the sensitivity of detection of SGA infants. However, the specificity was higher for selective ultrasonography (98%) than universal ultrasonography (90%). After the absolute numbers of true and false positives were calculated, our findings showed that for every additional SGA infant correctly identified by universal ultrasonography, about two additional results were false positives.

On the basis of these results, implementation of universal ultrasonographic screening would likely increase the detection of SGA infants. However, it would also substantially increase the number of false positive results. The net effect on clinical outcomes would depend on the balance between any benefits that arise from identification of true positives versus any harm caused by false positives. However, even correct identification of SGA infants has the potential to cause unnecessary intervention. The population of SGA infants is well recognised to consist of both those that are healthy but small in size and those with restricted growth. We postulated that effective markers of growth restriction would identify the small fetuses who were at increased risk of neonatal morbidity and assessed five previously described ultrasonographic markers of FGR (figure 2). The only measurement that had strong evidence for an interaction was the fetal abdominal circumference growth velocity. In all other cases, SGA infants were still at increased risk of morbidity if the indicator of FGR was normal. By contrast, an EFW of less than the 10th percentile was not associated with neonatal morbidity if the infant's abdominal circumference growth velocity was normal, but was associated with about a four times increased risk of neonatal morbidity if the abdominal circumference growth velocity was in the lowest decile. Furthermore, combination of an EFW of less than the 10th percentile plus an abdominal circumference growth velocity in the lowest decile was associated with about an 18 times increased risk of mothers delivering an SGA infant with neonatal morbidity, and about a 40 times increased risk of mothers delivering an SGA infant with a severe adverse perinatal outcome. We repeated the analyses using the 2014 international reference standard to quantify abdominal circumference growth velocity^25^ and the results were largely identical.

Our results showed that customisation of the EFW did not increase the strength of association between SGA and neonatal morbidity. The process of customisation attempts to relate the estimated size of a fetus to its genetic potential, using the maternal characteristics. We interpreted the findings of this study to suggest that the size of a fetus at the 20 week scan might be a better proxy of its genetic growth potential than the maternal characteristics. Our finding that abdominal circumference growth velocity was better than either uterine or umbilical Doppler to distinguish between SGA infants at low risk and high risk is consistent with the view that poor growth could be an endpoint of several pathological changes. Hence, assessment of growth velocity might be a more appropriate marker of adverse outcome as additional specific tests only provide information about a subset of FGR caused by a specific pathophysiological pathway.

Our study has strengths and weaknesses. One of the strengths is that clinicians were blinded to the results of research ultrasonographic assessments of fetal biometry and uteroplacental Doppler. The justification for concealment of the results of the research biometry and uteroplacental Doppler was taken from the NICE recommendation that these scans should not be offered routinely.^3^ The rationale for concealment of the biometry was that if the results had been known, they might have biased subsequent assessment of symphyseal-fundal height. A limitation of our study is that it was confined to nulliparous women. The rationale for selection of this group was that nulliparous women have higher rates of SGA than multiparous women and, by definition, no information is available about any history of previous SGA births in this group, which is one of the strongest predictors of SGA in a pregnancy. However, further studies are needed to establish whether universal ultrasonography is also effective in multiparous women. The major single cause of non-anomalous perinatal deaths at term is antepartum stillbirth, and about 30% of these stillbirths are associated with poor fetal growth.^32^ Although stillbirth was included in our composite of severe adverse outcome, our study was underpowered to investigate stillbirth directly. However, we speculate that the same ultrasonic features associated with neonatal morbidity and the composite of severe adverse perinatal outcome are likely to be associated with the risk of stillbirth.

In conclusion, we showed that universal third trimester ultrasound tripled the detection of SGA infants and could identify FGR fetuses that were at increased risk of neonatal morbidity. The guideline^1^ from the Royal College of Obstetricians and Gynaecologists (RCOG) lists a series of evidence-based recommendations for the management of suspected FGR—including fetal monitoring, timing of induction of labour, and how to undertake delivery.^1^ We believe that a programme of screening that includes universal ultrasonography and intervention following a care bundle based on the latest RCOG guideline^1^ has the potential to reduce the number of adverse perinatal outcomes caused by FGR.

**This online publication has been corrected. The corrected version first appeared at thelancet.com on November 19, 2015**

## Figures and Tables

**Table 1:** Characteristics of the study cohort

		**No clinically indicated scan ≥26 weeks (n=2311)**	**One or more clinically indicated scan ≥26 weeks (n=1666)**	**p value**	**Overall baseline characteristics (N=3977)**
**Maternal characteristics**					
Age (years)				<0·0001	
	<20	66 (3%)	73 (4%)	..	139 (4%)
	20–24·9	311 (13%)	209 (13%)	..	520 (13%)
	25–29·9	757 (33%)	468 (28%)	..	1225 (31%)
	30–34·9	887 (38%)	598 (36%)	..	1485 (37%)
	35–39·9	274 (12%)	260 (16%)	..	534 (13%)
	≥40	16 (1%)	58 (3%)	..	74 (2%)
Age stopped FTE (years)				0·01	
	<19	728 (32%)	593 (36%)	..	1321 (33%)
	19–22	828 (36%)	555 (33%)	..	1383 (35%)
	≥23	697 (30%)	463 (28%)	..	1160 (29%)
	Missing	58 (3%)	55 (3%)	NA	113 (3%)
Deprivation quartile				0·18	
	1 (lowest)	574 (25%)	400 (24%)	..	974 (24%)
	2	550 (24%)	393 (24%)	..	943 (24%)
	3	561 (24%)	399 (24%)	..	960 (24%)
	4 (highest)	523 (23%)	416 (25%)	..	939 (24%)
	Missing	103 (4%)	58 (3%)	NA	161 (4%)
Postcode area				0·17	
	CB1–5	709 (31%)	514 (31%)	..	1223 (31%)
	CB21–25	528 (23%)	374 (22%)	..	902 (23%)
	CB6–11	549 (24%)	446 (27%)	..	995 (25%)
	Outside Cambridgeshire	459 (20%)	302 (18%)	..	761 (19%)
	Missing	66 (3%)	30 (2%)	NA	96 (2%)
Ethnic origin					
	White	2151 (93%)	1545 (93%)	0·65	3696 (93%)
	Missing	40 (2%)	29 (2%)	NA	69 (2%)
Married		1576 (68%)	1151 (69%)	0·55	2727 (69%)
Smoker		106 (5%)	79 (5%)	0·82	185 (5%)
Alcohol consumption					
	Any	117 (5%)	66 (4%)	0·10	183 (5%)
	Missing	1 (<1%)	0 (0%)	NA	1 (<1%)
BMI (kg/m^2^)				<0·0001	
	<25	1416 (61%)	909 (55%)	..	2325 (58%)
	25–29·9	667 (29%)	450 (27%)	..	1117 (28%)
	30–34·9	209 (9%)	168 (10%)	..	377 (9%)
	35–39·9	18 (1%)	92 (6%)	..	110 (3%)
	≥40	1 (<1%)	46 (3%)	..	47 (1%)
	Missing	0 (0%)	1 (<1%)	NA	1 (<1%)
≥1 previous miscarriage		207 (9%)	199 (12%)	0·002	406 (10%)
Diabetes				<0·0001	
	Type 1 or type 2	0 (0%)	14 (1%)	..	14 (<1%)
	Gestational	2 (<1%)	160 (10%)	..	162 (4%)
	Missing	3 (<1%)	2 (<1%)	NA	5 (<1%)
**Birth outcomes**					
Birthweight (g)		3480 (3175–3770)	3345 (3010–3685)	<0·0001	3420 (3105–3740)
SGA (<10th)		178 (8%)	174 (10%)	0·003	352 (9%)
Severe SGA (<3rd)		34 (1%)	53 (3%)	0·0003	87 (2%)
Gestational age (weeks)				<0·0001	
	Preterm (26–32)	15 (1%)	15 (1%)	..	30 (1%)
	Preterm (33–36)	53 (2%)	80 (5%)	..	133 (3%)
	Term (≥37)	2243 (97%)	1571 (94%)	..	3814 (96%)
Induction of labour					
	Yes	629 (27%)	629 (38%)	<0·0001	1258 (32%)
	Missing	4 (<1%)	2 (<1%)	NA	6 (<1%)
Mode of delivery				<0·0001	
	Spontaneous vaginal	1218 (53%)	706 (42%)	..	1924 (48%)
	Assisted vaginal	596 (26%)	353 (21%)	..	949 (24%)
	Intrapartum caesarean	415 (18%)	283 (17%)	..	698 (18%)
	Prelabour caesarean	74 (3%)	317 (19%)	..	391 (10%)
	Missing	8 (<1%)	7 (<1%)	NA	15 (<1%)

Data are n (%) or median (IQR). p values are for difference between groups calculated using the two-sample Wilcoxon rank-sum (Mann-Whitney) test for continuous variables and the Pearson χ^2^ test for categorical variables, with trend tests if appropriate. The “missing” category was not included in statistical tests. For characteristics that have no “missing” category, data were 100% complete. Maternal age was defined as age at recruitment to study. All other maternal characteristics were defined by self-report at the 20 weeks questionnaire, from examination of the clinical case record, or linkage to the hospital's electronic databases. Socioeconomic status was quantified by use of the Index of Multiple Deprivation^24^ 2007, which is based on census data from the area of the mother's postcode. FTE=full-time education. CB1–5=central Cambridge city. CB21–25=peripheral Cambridge city. CB6–11=Cambridgeshire, outside city. NA=not applicable. BMI=body-mass index. SGA=small for gestational age.

**Table 2:** Screening effectiveness for selective and universal ultrasonographic screening for infants who are small and severely small for gestational age

	**SGA**			**Severe SGA**		
	Yes	No	Total	Yes	No	Total
**Selective ultrasonography**						
EFW <10th	69	69	138	28	110	138
EFW ≥10th or no scan	283	3556	3839	59	3780	3839
Total	352	3625	3977	87	3890	3977
**Universal ultrasonography**						
EFW <10th	199	363	562	67	495	562
EFW ≥10th	153	3262	3415	20	3395	3415
Total	352	3625	3977	87	3890	3977

Selective ultrasonography shows the results of clinically indicated scans. Of the 1666 women selected for ultrasonography at 26 weeks or later, 1388 (83%) had one or two scans, 245 (15%) had three or four scans, and 33 (2%) had five or more scans. If a woman did not have a clinically indicated scan after the routine anomaly scan she was defined as screen negative by selective ultrasonography. Universal ultrasonography shows the results of the last research scan done before birth (either the 28 week scan or the 36 week scan, depending on the gestational age at delivery). Median time interval (IQR) between the last selective scan and birth was 3·1 weeks (1·6–5·6 weeks), and between the last universal scan and birth was 4·1 weeks (3·1–5·0 weeks). SGA=small for gestational age (birthweight <10th percentile; severe SGA birthweight <3rd percentile). EFW=estimated fetal weight (from the last scan before birth).

**Table 3:** Diagnostic effectiveness of selective versus universal ultrasonographic screening for infants who are small and severely small for gestational age

	**SGA**		**Severe SGA**	
	Selective	Universal	Selective	Universal
Sensitivity (%)	20% (15–24)	57% (51–62)	32% (22–42)	77% (68–86)
Specificity (%)	98% (98–99)	90% (89–91)	97% (97–98)	87% (86–88)
Positive predictive value (%)	50% (42–58)	35% (31–39)	20% (14–27)	12% (9–15)
Negative predictive value (%)	93% (92–93)	96% (95–96)	98% (98–99)	99% (99–100)
False positive rate* (%)	2% (1–2)	10% (9–11)	3% (2–3)	13% (12–14)
False negative rate† (%)	80% (76–85)	43% (38–49)	68% (58–78)	23% (14–32)
Positive likelihood ratio	10·3 (7·5–14·1)	5·6 (4·9–6·5)‡	11·4 (8·0–16·3)	6·1 (5·3–7·0)
Negative likelihood ratio	0·8 (0·8–0·9)	0·5 (0·4–0·5)‡	0·7 (0·6–0·8)	0·3 (0·2–0·4)
Relative sensitivity	1·0 (reference)	2·9 (2·4–3·5)	1·0 (reference)	2·4 (1·8–3·2)

95% CIs are given in brackets. All values were calculated with estimated fetal weight <10th percentile as screen positive. Statistical comparison by McNemar, weighted generalised score tests, or regression model-based tests as appropriate. All comparisons of selective *vs* universal had p<0·0001 for both outcomes, except for SGA positive likelihood ratio (p=0·0001), severe SGA positive predictive value (p=0·0002), and positive likelihood ratio (p=0·0003). SGA=small for gestational age (birthweight <10th percentile; severe SGA birthweight <3rd percentile).

* Defined as proportion of screen positives among non-SGA infants.

† Defined as proportion of screen negatives among SGA infants.

‡ Sample calculation: positive likelihood ratio= (199 ÷ 363)/(352 ÷ 3625)=5·6; negative likelihood ratio= (153 ÷ 3262)/(352 ÷ 3625)=0·5.

**Table 4:** Association between perinatal outcomes of estimated fetal weight less than the 10th percentile and abdominal circumference growth velocity

	**Any neonatal morbidity (n=275)**		**Metabolic acidosis (n=42)**		**5 min Apgar <7 (n=36)**		**Neonatal unit admission (n=229)**		**SGA plus any neonatal morbidity (n=49)**		**Severe adverse perinatal outcome (n=33)**		**SGA plus severe adverse perinatal outcome (n=5)**	
	RR (95% CI)	p value	RR (95% CI)	p value	RR (95% CI)	p value	RR (95% CI)	p value	RR (95% CI)	p value	RR (95% CI)	p value	RR (95% CI)	p value
EFW <10th (population)	1·6 (1·2–2·1)	0·001	1·4 (0·7–3·1)	0·37	2·3 (1.1–4·8)	0·03	1·6 (1·2–2·1)	0·006	10·5 (5·9–18·6)	<0·0001	1·4 (0·6–3·3)	0·45	24·3 (2·7–217·1)	0·002
EFW <10th (customised*)	1·7 (1·3–2·3)	0·001	1·5 (0·6–3·4)	0·44	1·7 (0·7–4.2)	0·26	1·6 (1·2–2·3)	0·01	9·8 (5·7–17·1)	<0·0001	1·9 (0·8–4·7)	0·14	34·8 (3·9–310·4)	0·0005
EFW <10th plus normal ACGV	1·3 (0·9–1·8)	0·23	0·3 (0·0–1·9)	0·25	1·4 (0.5–3·9)	0·54	1·4 (0·9–2·0)	0·13	7·3 (3·7–14·4)	<0·0001	0·7 (0·2–2·7)	0·76	17·6 (1·6–193·5)	0·03
EFW <10th plus lowest decile ACGV	2·5 (1·7–3·6)	<0·0001	4·1 (1·8–9·1)	0·003	4·6 (1·9–11·0)	0·004	2·1 (1·3–3·2)	0·003	17·6 (9·2–34·0)	<0·0001	2·9 (1·0–8·3)	0·06	39·8 (3·6–436·6)	0·007

All estimated fetal weights (EFWs) are based on population-based percentiles, unless stated otherwise. All relative risks (RRs) are referent to infants with an EFW of ≥10th percentile by population-based standards, except for the RRs for customised EFW <10th percentile, which are referent to infants with an EFW of the ≥10th percentile by customised standards. Appendix has n/N for every cell. Small for gestational age (SGA) is defined as birthweight of <10th percentile by population standards. Abdominal circumference growth velocity (ACGV) is based on the change in the gestational age adjusted *Z* score, comparing the result at the 20 week scan with the last scan before birth. Neonatal morbidity is a composite outcome—ie, one or more of these three outcomes: metabolic acidosis (defined as pH <7·1 and base deficit >10 mmol/L), 5 min Apgar score less than 7, and neonatal unit admission (defined as admission to the neonatal intensive care unit, the high dependency unit, or the special care baby unit). Severe adverse perinatal outcome is a composite outcome—ie, one or more of the following outcomes specified: stillbirth (not due to congenital anomaly), neonatal death at term (not due to congenital anomaly), hypoxic ischaemic encephalopathy at term, use of inotropes at term, mechanical ventilation at term, severe metabolic acidosis at term (defined as pH <7·0 and base deficit >12 mmol/L). p values (all two sided) are from Fisher's exact test.

* Customised percentiles of EFW were calculated with the Gestation-Related Optimal Weight Customised Weight Centile Calculator (version 6.7 [UK]).

## Panel Research in context

**Systematic review**

Previously reported large-scale systematic reviews have addressed the ability of universal ultrasonography to result in improved clinical outcome. Reported Cochrane reviews have examined both conventional ultrasound and use of Doppler flow velocimetry of the uteroplacental circulation. Neither of these methods has been shown to improve outcome. The National Institute for Health and Care Excellence (NICE) did a highly detailed review of the methods of screening women at low risk for fetal wellbeing for the 2008 Guideline on Antenatal Care. This guideline included both results of the Cochrane reviews of clinical effectiveness of universal ultrasound and results of the new meta-analyses of diagnostic effectiveness of screening for small-for-gestational-age (SGA) infants. The guideline concluded that high quality evidence on the diagnostic effectiveness of routine ultrasonography was scarce, and recommended several aspects of screening for SGA infants as research priorities. It might seem paradoxical that universal ultrasonography has not been shown to be beneficial in randomised controlled trials, but NICE recommended further research into its diagnostic effectiveness. However, this absence of benefit might be attributed to the impossibility of designing an interventional study of screening without knowledge of the diagnostic effectiveness of the screening test in the given population. Moreover, many observational studies reported that SGA infants are at increased risk of complications, methods used to screen for SGA have low sensitivity and specificity, and undiagnosed SGA is a common finding in perinatal deaths.

**Interpretation**

Our study shows that universal use of ultrasound roughly tripled the detection of SGA infants. Moreover, by use of a combination of assessment of the estimated fetal weight of a baby with a previously reported measurement of fetal growth restriction (abdominal circumference growth velocity), the population of small fetuses could be divided into about 30% that were growth restricted and at increased risk of neonatal morbidity, and about 70% that did not have growth restriction and were at the same risk of neonatal morbidity as those babies not identified as small by universal ultrasound. The statistical associations (for both main effects and interactions) reported were sufficiently strong that these findings were unlikely to be chance or data driven. These results suggest that the screening of unselected nulliparous women and identification of growth-restricted fetuses at increased risk of an adverse outcome is possible. Additionally, the same combination of findings associated with the risk of neonatal morbidity could plausibly be predictive of other adverse outcomes associated with fetal growth restriction, such as antepartum stillbirth. As a result, a programme to screen with universal ultrasonography and intervention using a care bundle based on the latest Royal College of Obstetricians and Gynaecologists guideline might reduce the number of adverse perinatal outcomes caused by fetal growth restriction.
