# Department of Error

**DOI:** 10.1016/S0140-6736(15)00976-9

**Published:** 2015-11-21

*Sovio U, White IR, Dacey A, Pasupathy D, Smith GCS, Screening for fetal growth restriction with universal third trimester ultrasonography in nulliparous women in the Pregnancy Outcome Prediction (POP) study: a prospective cohort study.* Lancet *2015; **386:** 2089–97*—The Declaration of interests section should have read “DP reports a grant from the Medical Research Council (MRC), during the study. GCSS reports grants from the National Institute for Health Research (NIHR), Stillbirth and Neonatal Death Society, and MRC, and reports other from GE and NIHR Cambridge Clinical Research Facility, during the study; reports personal fees and other from GlaxoSmithKline and Roche, and reports other from Chiesi, outside of the study. US, IRW, and AD declare no competing interests.” These corrections have been made to the online version as of Nov 19, 2015, and the printed Article is correct.

