# Supplementary material for: Screening for fetal growth restriction with universal third trimester ultrasonography in nulliparous women in the Pregnancy Outcome Prediction (POP) study: a prospective cohort study
Source: Lancet. 2015 Nov 21;386(10008):2089–97. doi: 10.1016/S0140-6736(15)00131-2 (PMC4655320; doi:10.1016/S0140-6736(15)00131-2)
Supplement: Supplementary appendix [file mmc1.pdf]

THE LANCET

Supplementary appendix

This appendix formed part of the original submission and has been peer reviewed. We post it as supplied by the authors.

Supplement to: Sovio U, White IR, Dacey A, Pasupathy D, Smith GCS. Screening for fetal growth restriction with universal third trimester ultrasonography in nulliparous women in the Pregnancy Outcome Prediction (POP) study: a prospective cohort study. *Lancet* 2015; published online Sept 8. http://dx.doi.org/10.1016/S0140-6736(15)00131-2.

Supplementary Information for Sovio et al, Screening for fetal growth restriction using universal third trimester ultrasonography: a prospective cohort study of 3,977 nulliparous women.

Contents

Supplementary Figure 1. Measurement of fetal abdominal circumference at 20, 28 & 36 weeks.
Supplementary Figure 2. Measurement of fetal biparietal diameter at 20, 28 & 36 weeks.
Supplementary Figure 3. Measurement of fetal head circumference at 20, 28 & 36 weeks.
Supplementary Figure 4. Measurement of fetal femur length at 20, 28 & 36 weeks.
Supplementary Figure 5. Measurement of estimated fetal weight at 20, 28 & 36 weeks.
Supplementary Figure 6. Measurement of uterine artery mean pulsatility index at 20, 28 & 36 weeks.
Supplementary Figure 7. Measurement of umbilical artery mean pulsatility index at 20, 28 & 36 weeks.
Supplementary Figure 8. Scatter plot and Bland-Altman plot describing inter-observer repeatability in a sub-sample: fetal abdominal circumference (AC) at 20 weeks (n=45) and 36 weeks (n=44).
Supplementary Figure 9. Scatter plot and Bland-Altman plot describing inter-observer repeatability in a sub-sample: fetal biparietal diameter (BPD) at 20 weeks (n=45) and 36 weeks (n=36).
Supplementary Figure 10. Scatter plot and Bland-Altman plot describing inter-observer repeatability in a sub-sample: fetal head circumference (HC) at 20 weeks (n=45) and 36 weeks (n=37).
Supplementary Figure 11. Scatter plot and Bland-Altman plot describing inter-observer repeatability in a sub-sample: fetal femur length (FL) at 20 weeks (n=45) and 36 weeks (n=43).
Supplementary Figure 12. Scatter plot and Bland-Altman plot describing inter-observer repeatability in a sub-sample: estimated fetal weight (EFW) at 20 weeks (n=45) and 36 weeks (n=43).
Supplementary Figure 13. Scatter plot and Bland-Altman plot describing inter-observer repeatability in a sub-sample: uterine artery mean pulsatility index (UTPI) at 20 weeks (n=44) and 36 weeks (n=42).
Supplementary Figure 14. Scatter plot and Bland-Altman plot describing inter-observer repeatability in a sub-sample: umbilical artery mean pulsatility index (UMPI) at 20 weeks (n=45) and 36 weeks (n=43).
Supplementary Figure 15. Receiver operating characteristic curve analysis of universal and selective ultrasonography.
Supplementary Figure 16. Receiver operating characteristic curve analysis of universal ultrasonography, 28 week scan.
Supplementary Figure 17. Receiver operating characteristic curve analysis of universal ultrasonography, 36 week scan.
Supplementary Table 1. Equations for mean and standard deviation (SD) estimated within the POP study (n=3,977) by gestational age interval.
Supplementary Table 2. Comparisons of ultrasonographic measurements in the POP study with published reference values.
Supplementary Table 3. Analysis of reproducibility and reliability of measurements at 20 and 36 weeks gestational age.
Supplementary Table 4. Hospital record data comparing women recruited to the study with eligible women who were not recruited.
Supplementary Table 5. The area under the ROC curve (95% confidence interval) for universal ultrasonographic screening for SGA and severe SGA infants, stratified by gestational age at birth.
Supplementary Table 6. Sensitivity analyses for comparison of universal versus selective ultrasonography for detection of SGA (birth weight <10th percentile) infants.
Supplementary Table 7. Sensitivity analyses for comparison of universal versus selective ultrasonography for detection of severe SGA (birth weight <3rd percentile) infants.
Supplementary Table 8. The relationship between EFW<10th percentile, abdominal circumference growth velocity (derived from an international reference standard) and perinatal outcome.
Supplementary Table 9. The relationship between indicators of fetal growth restriction (FGR) and perinatal outcome in babies not diagnosed SGA.
Supplementary Table 10. Numbers (%) of perinatal outcomes by EFW<10th percentile and abdominal circumference growth velocity (ACGV).
Supplementary References

Supplementary Figure 1. Measurement of fetal abdominal circumference at 20, 28 & 36 weeks.

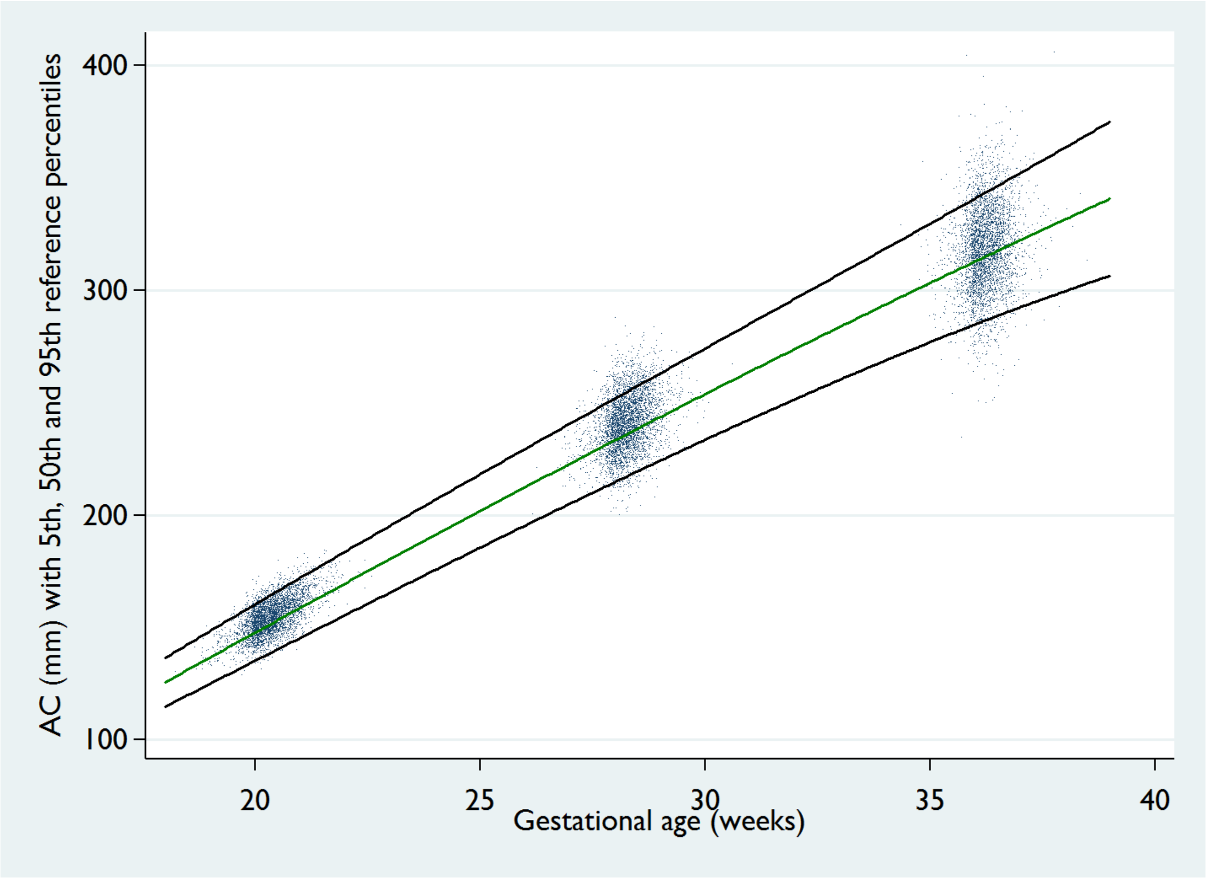

Plot of measurements of abdominal circumference (AC) measured in millimeters (mm) at the time of the clinical (reported) 20 week measurement and the research measurements at ~28 and ~36 weeks. The green line is the 50th percentile and the black lines above and below are the 5th and 95th percentiles of a previously published reference range.[1]

Supplementary Figure 2. Measurement of fetal biparietal diameter at 20, 28 & 36 weeks.

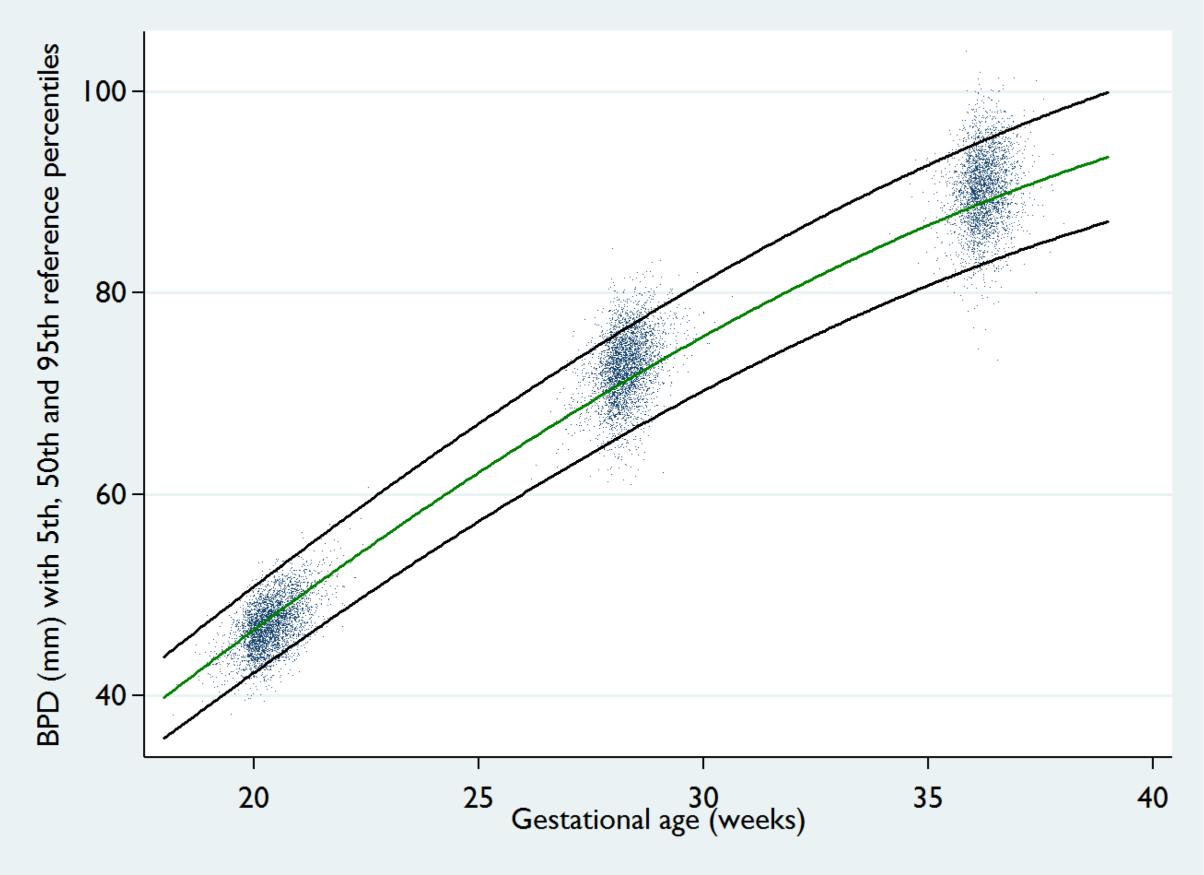

Plot of measurements of biparietal diameter (BPD) measured in millimeters (mm) at the time of the clinical (reported) 20 week measurement and the research measurements (blinded) at ~28 and ~36 weeks. The green line is the 50th percentile and the black lines above and below are the 5th and 95th percentiles of a previously published reference range.[2]

Supplementary Figure 3. Measurement of fetal head circumference at 20, 28 & 36 weeks.

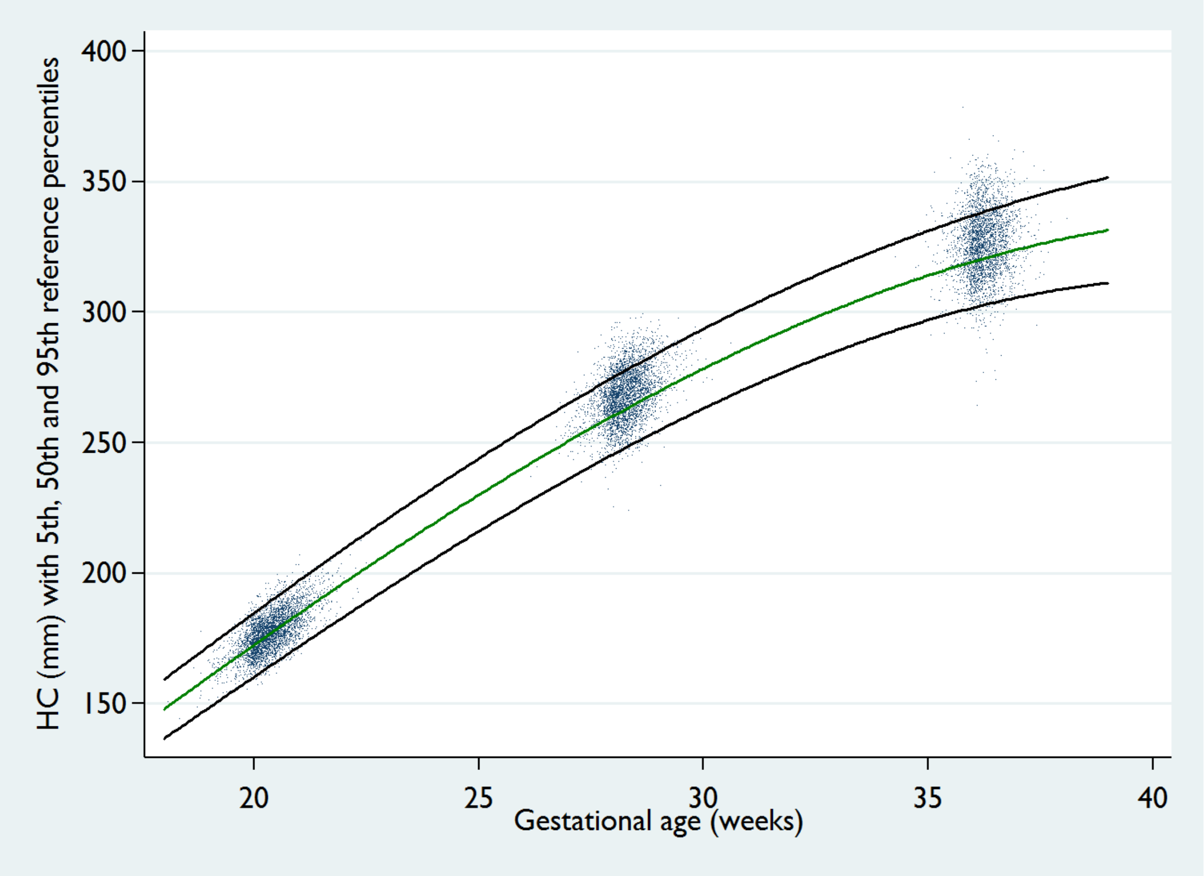

Plot of measurements of head circumference (HC) measured in millimeters (mm) at the time of the clinical (reported) 20 week measurement and the research measurements (blinded) at ~28 and ~36 weeks. The green line is the 50th percentile and the black lines above and below are the 5th and 95th percentiles of a previously published reference range.[1]

Supplementary Figure 4. Measurement of fetal femur length at 20, 28 & 36 weeks.

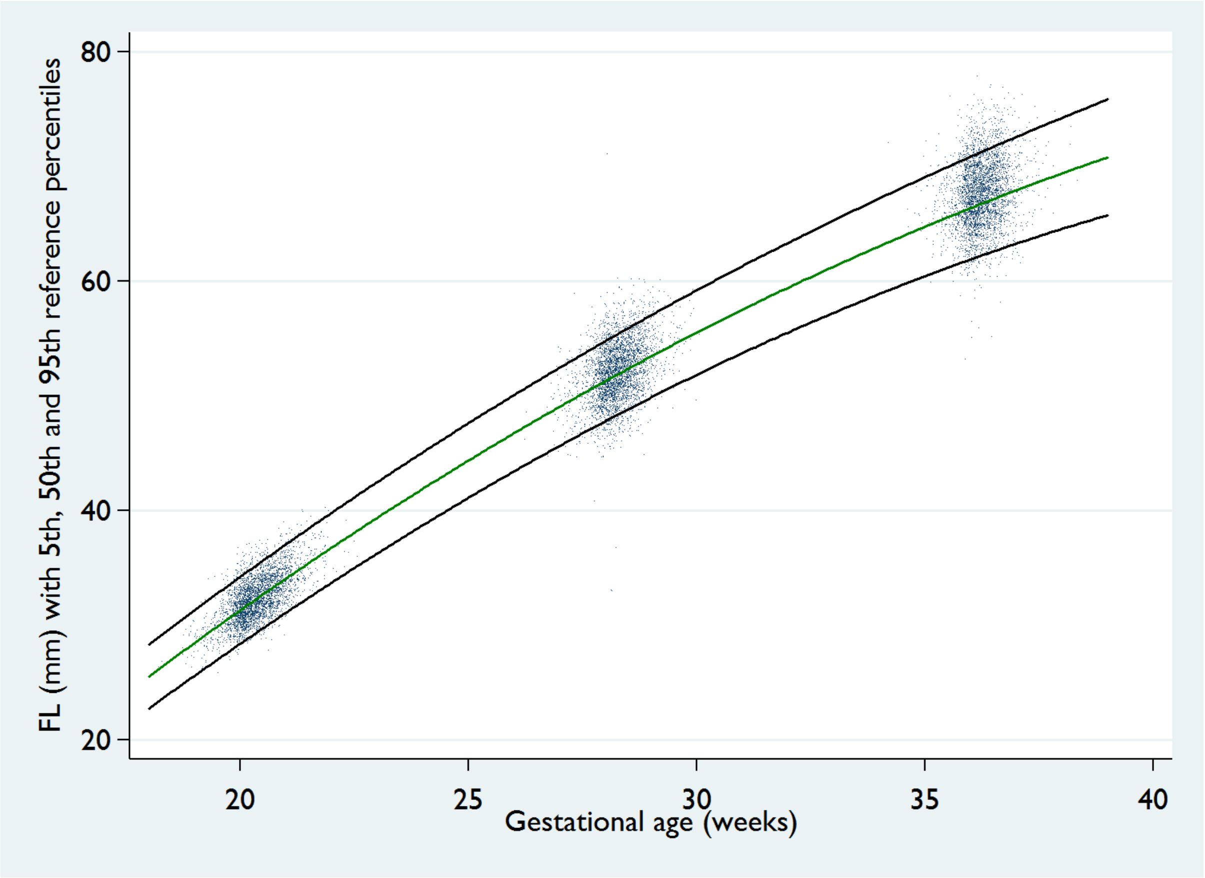

Plot of measurements of femur length (FL) measured in millimeters (mm) at the time of the clinical (reported) 20 week measurement and the research measurements (blinded) at ~28 and ~36 weeks. The green line is the 50th percentile and the black lines above and below are the 5th and 95th percentiles of a previously published reference range.[1]

Supplementary Figure 5. Measurement of estimated fetal weight at 20, 28 & 36 weeks.

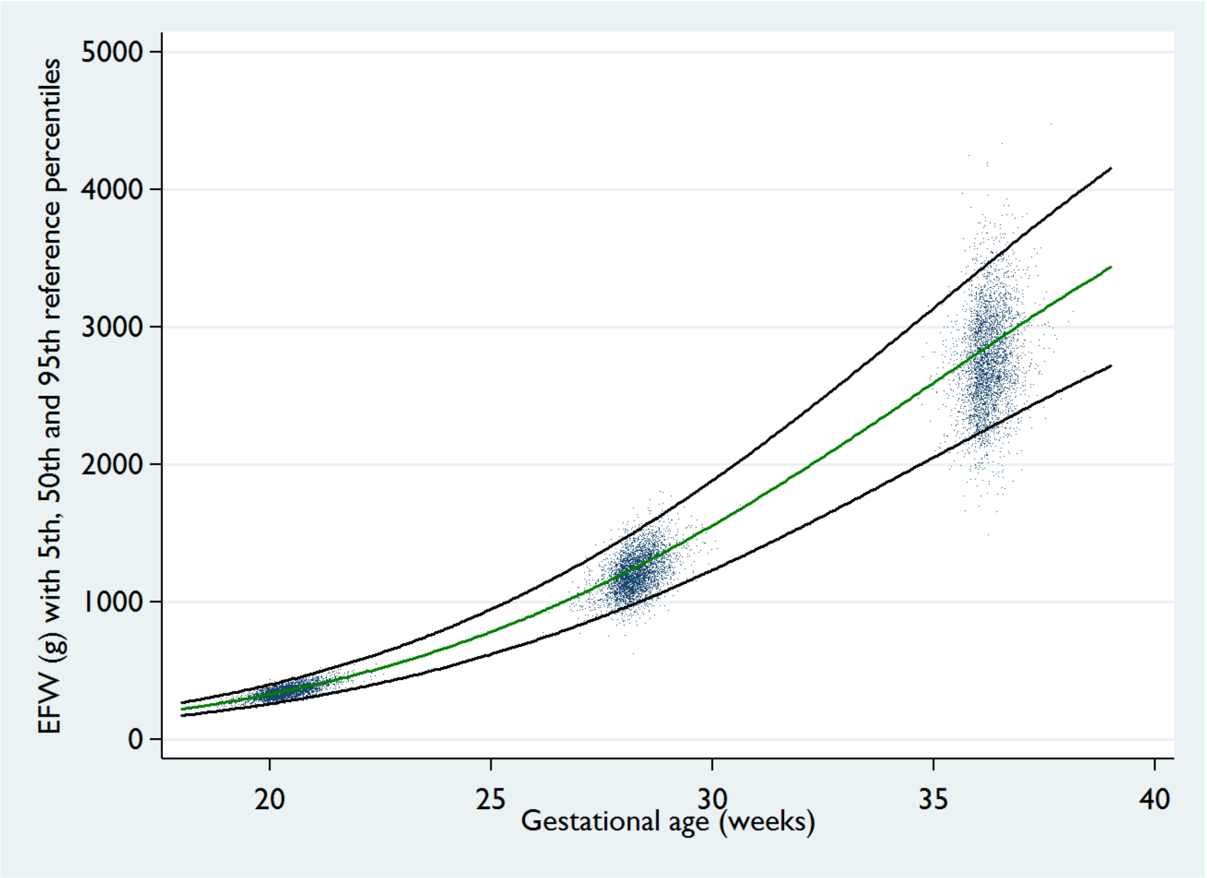

Plot of measurements of estimated fetal weight (EFW), calculated using the Hadlock et al (1985) equations,[3] at the time of the clinical (reported) 20 week measurement and the research measurements (blinded) at ~28 and ~36 weeks. The green line is the 50th percentile and the black lines above and below are the 5th and 95th percentiles of a previously published reference range.[4]

Supplementary Figure 6. Measurement of uterine artery mean pulsatility index at 20, 28 & 36 weeks.

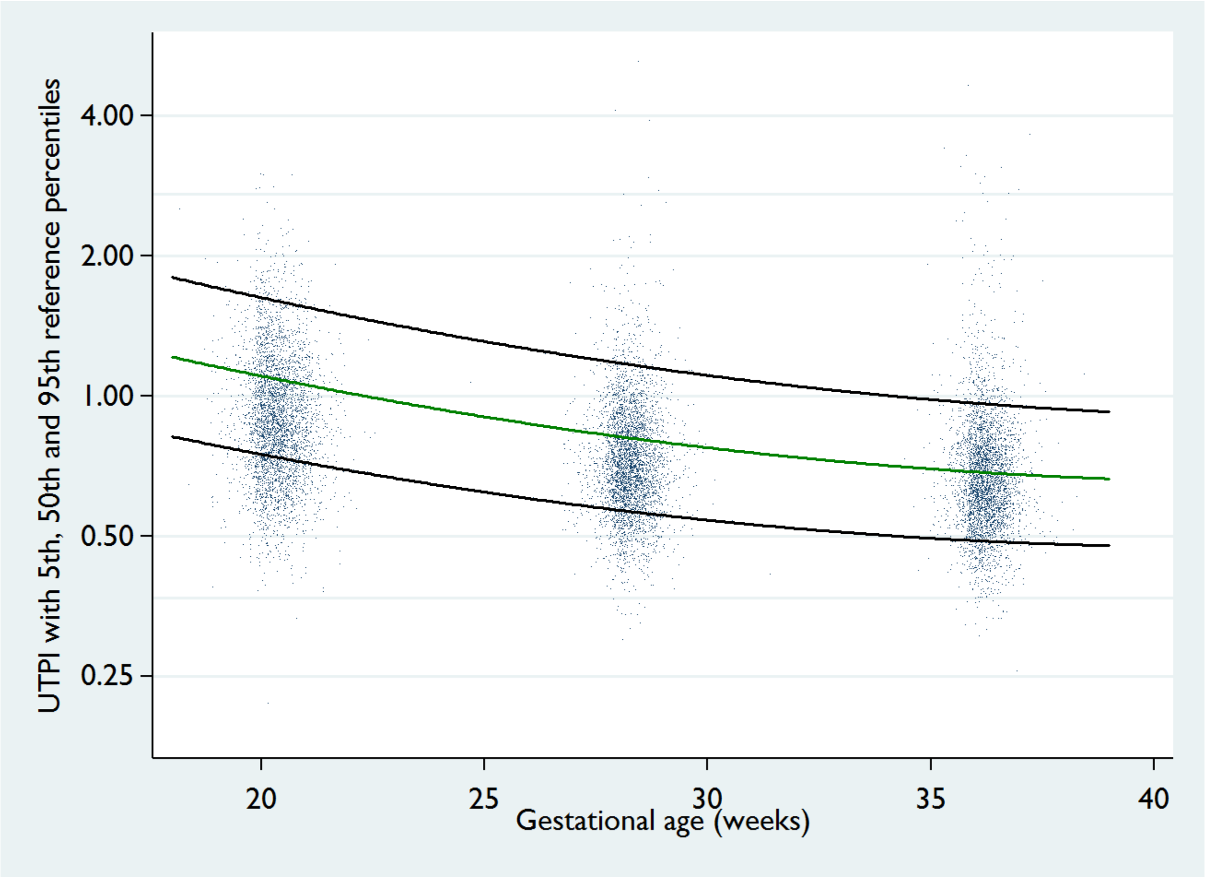

Plot of uterine artery mean pulsatility index (UTPI) measured for the purposes of research (blinded) at ~20, ~28 and ~36 weeks. The green line is the 50th percentile and the black lines above and below are the 5th and 95th percentiles of a previously published reference range. The values are plotted on a logarithmic scale.

Supplementary Figure 7. Measurement of umbilical artery pulsatility index at 20, 28 & 36 weeks.

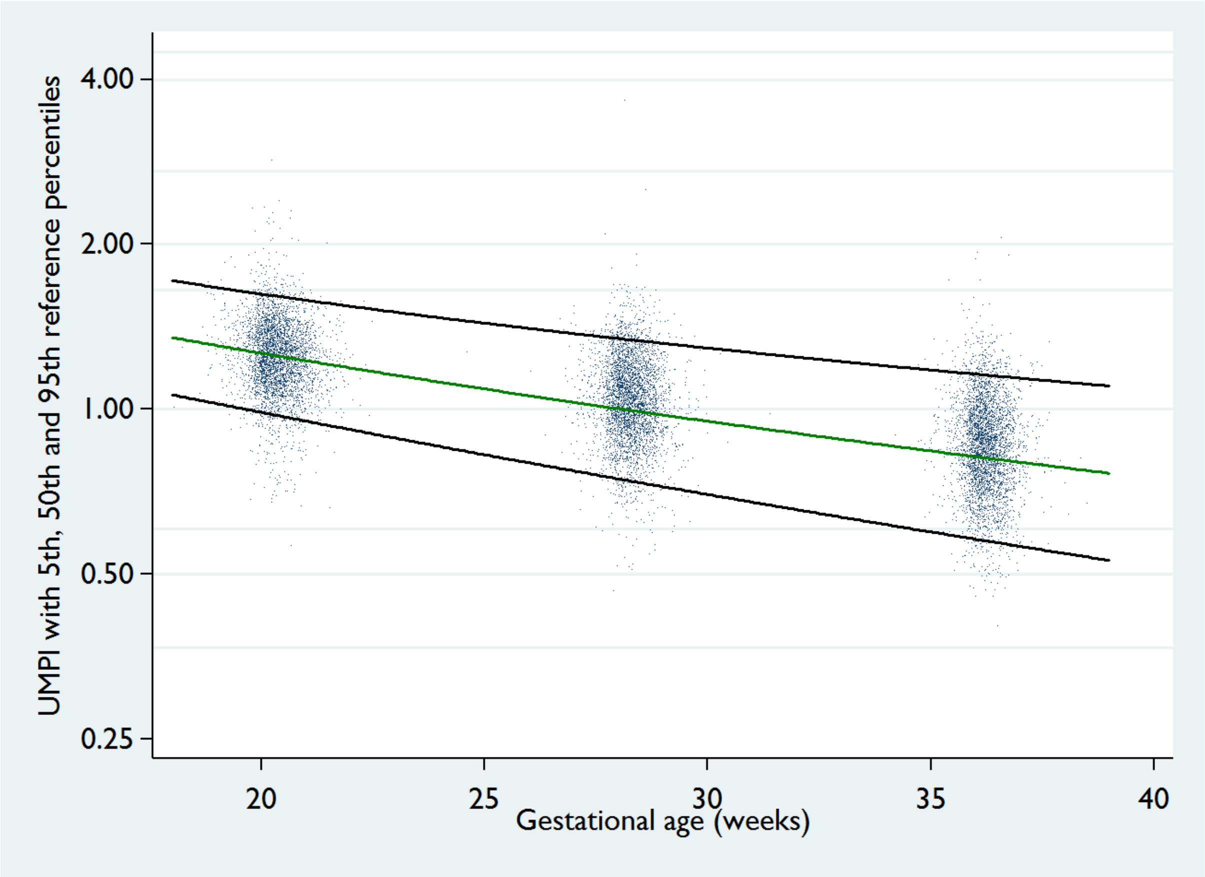

Plot of umbilical artery pulsatility index (UMPI) measured for the purposes of research (blinded) at ~20, ~28 and ~36 weeks. The green line is the 50th percentile and the black lines above and below are the 5th and 95th percentiles of a previously published reference range. The values are plotted on a logarithmic scale.

Supplementary Figure 8. Scatter plot and Bland-Altman plot describing inter-observer repeatability in a sub-sample: fetal abdominal circumference (AC) at 20 weeks (n=45) and 36 weeks (n=44).

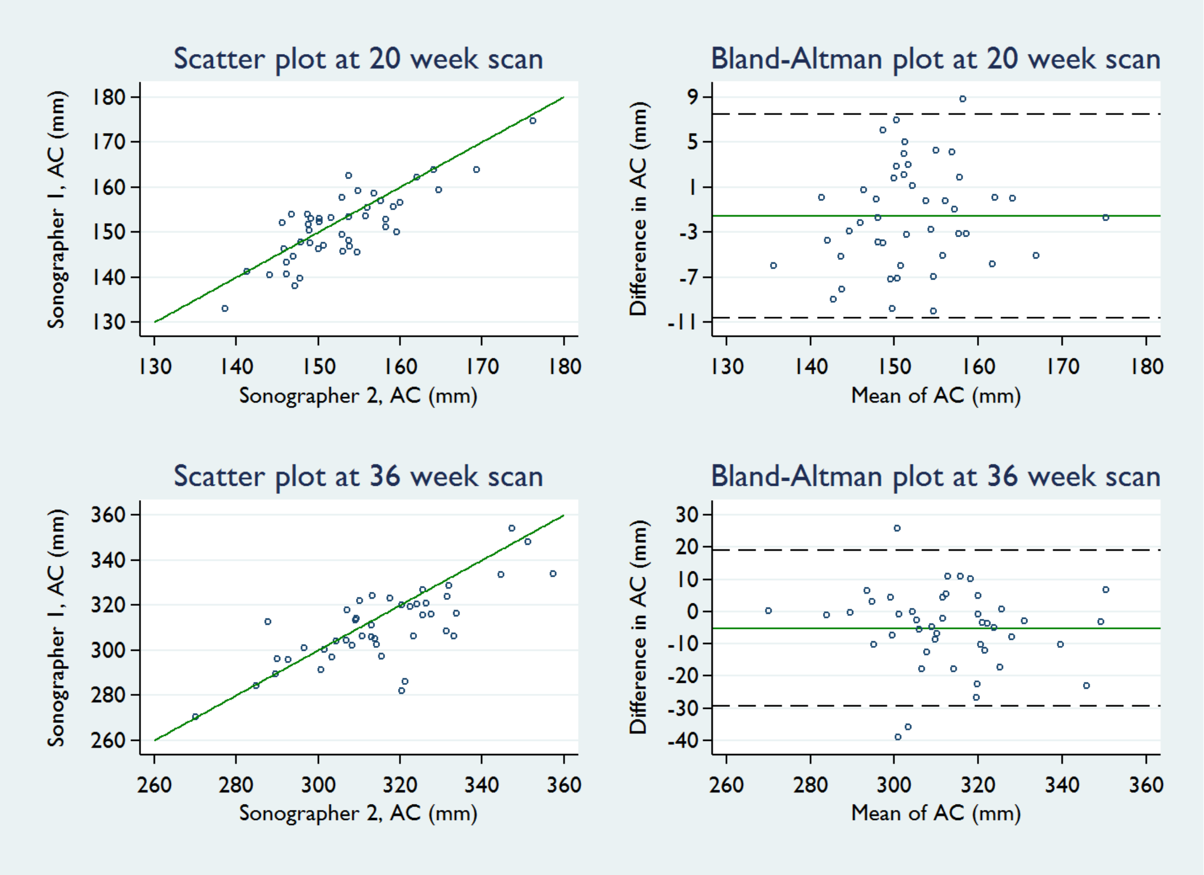

For the concordance correlation coefficient and 95% limits of agreement, refer to Supplementary Table 3.

Supplementary Figure 9. Scatter plot and Bland-Altman plot describing inter-observer repeatability in a sub-sample: fetal biparietal diameter (BPD) at 20 weeks (n=45) and 36 weeks (n=36).

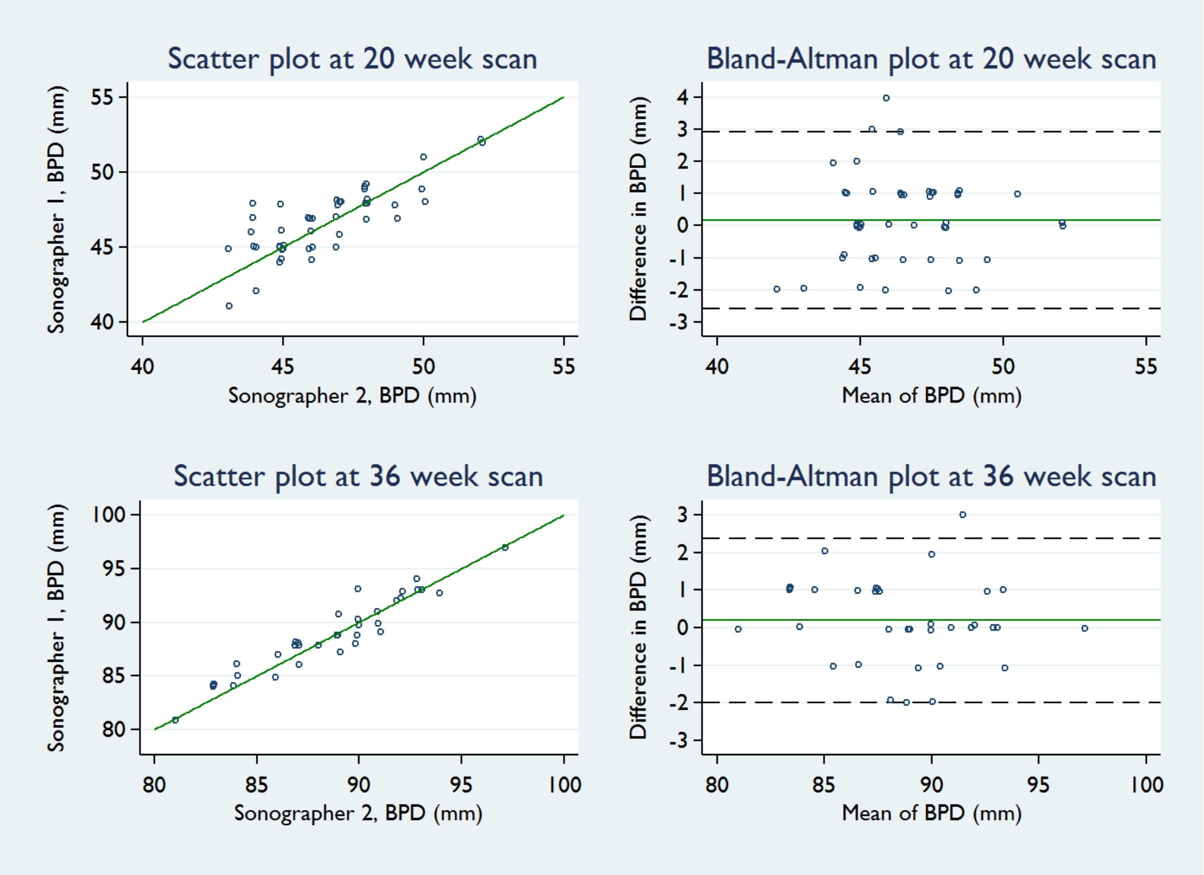

For the concordance correlation coefficient and 95% limits of agreement, refer to Supplementary Table 3.

Supplementary Figure 10. Scatter plot and Bland-Altman plot describing inter-observer repeatability in a sub-sample: fetal head circumference (HC) at 20 weeks (n=45) and 36 weeks (n=37).

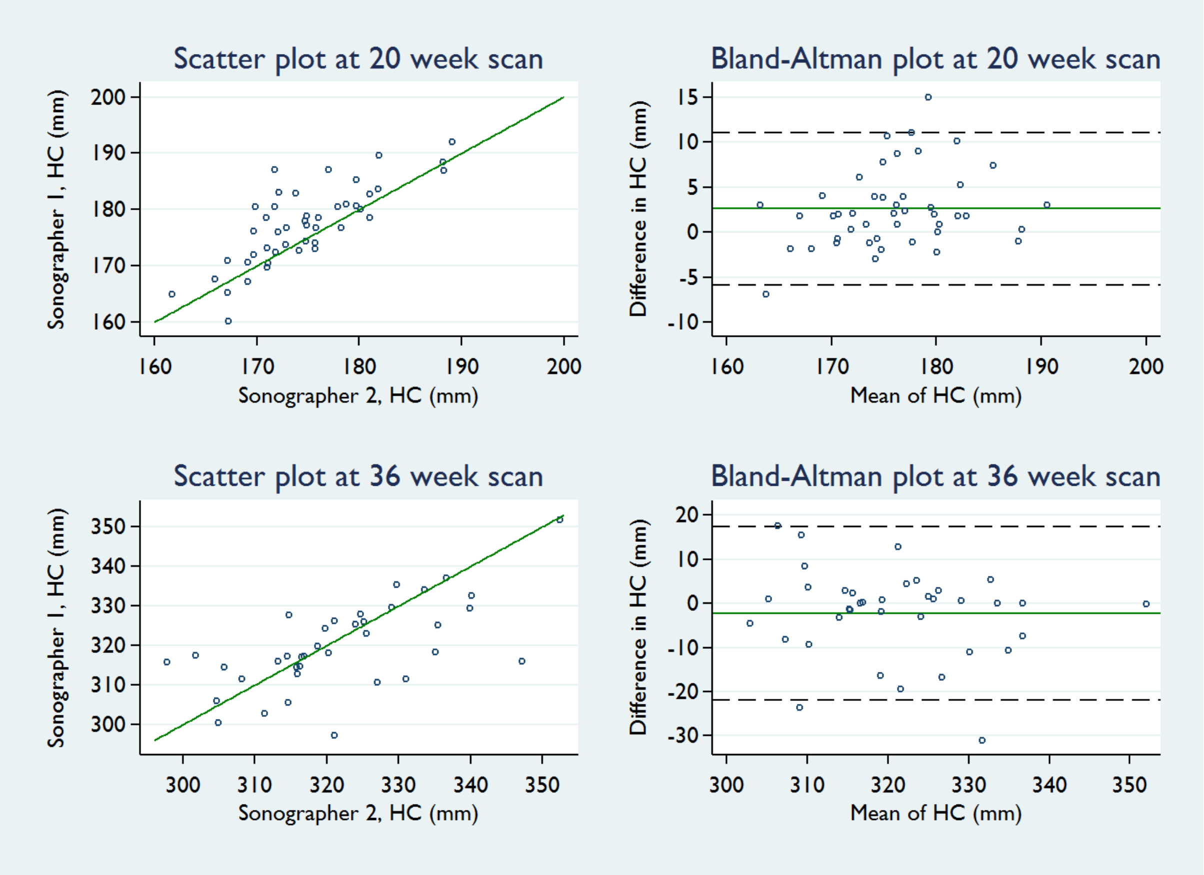

For the concordance correlation coefficient and 95% limits of agreement, refer to Supplementary Table 3.

Supplementary Figure 11. Scatter plot and Bland-Altman plot describing inter-observer repeatability in a sub-sample: fetal femur length (FL) at 20 weeks (n=45) and 36 weeks (n=43).

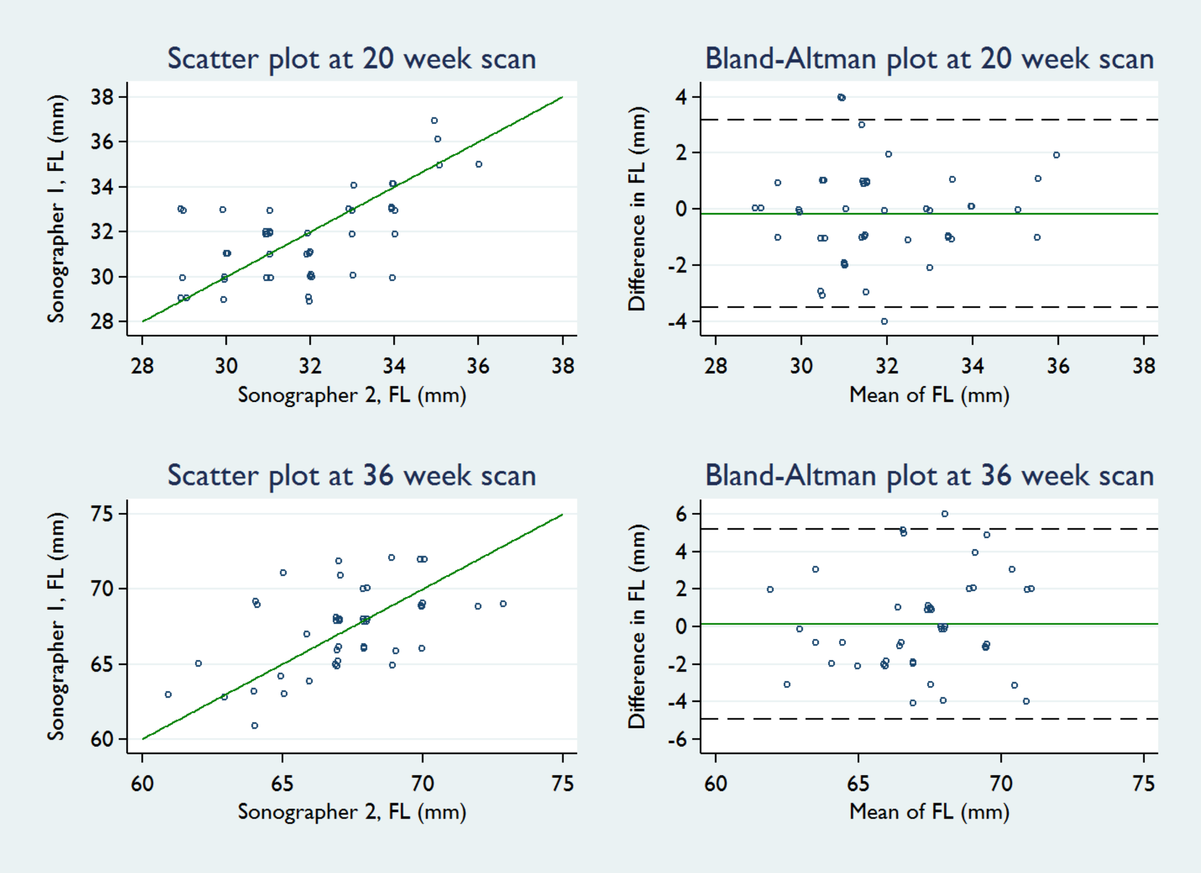

For the concordance correlation coefficient and 95% limits of agreement, refer to Supplementary Table 3.

Supplementary Figure 12. Scatter plot and Bland-Altman plot describing inter-observer repeatability in a sub-sample: estimated fetal weight (EFW) at 20 weeks (n=45) and 36 weeks (n=43).

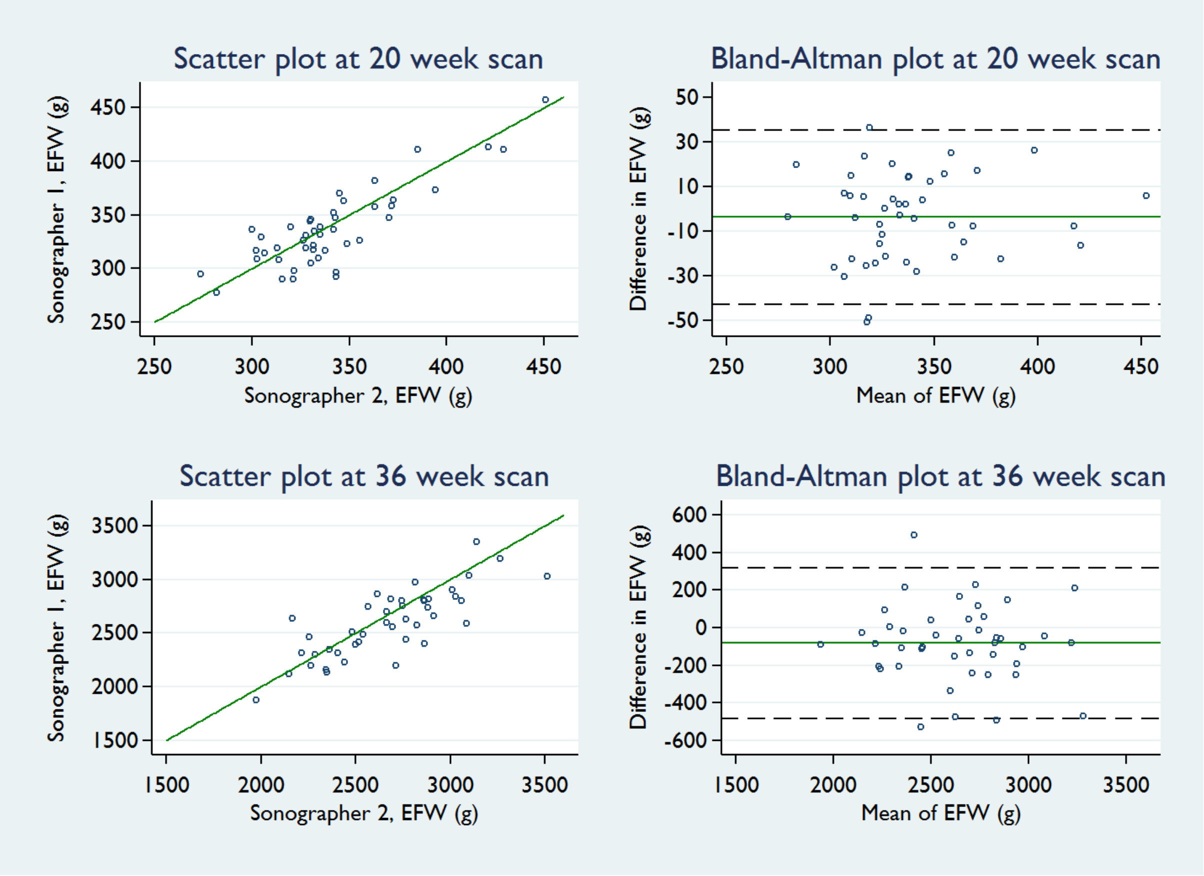

For the concordance correlation coefficient and 95% limits of agreement, refer to Supplementary Table 3.

Supplementary Figure 13. Scatter plot and Bland-Altman plot describing inter-observer repeatability in a sub-sample: uterine artery mean pulsatility index (UTPI) at 20 weeks (n=44) and 36 weeks (n=42).

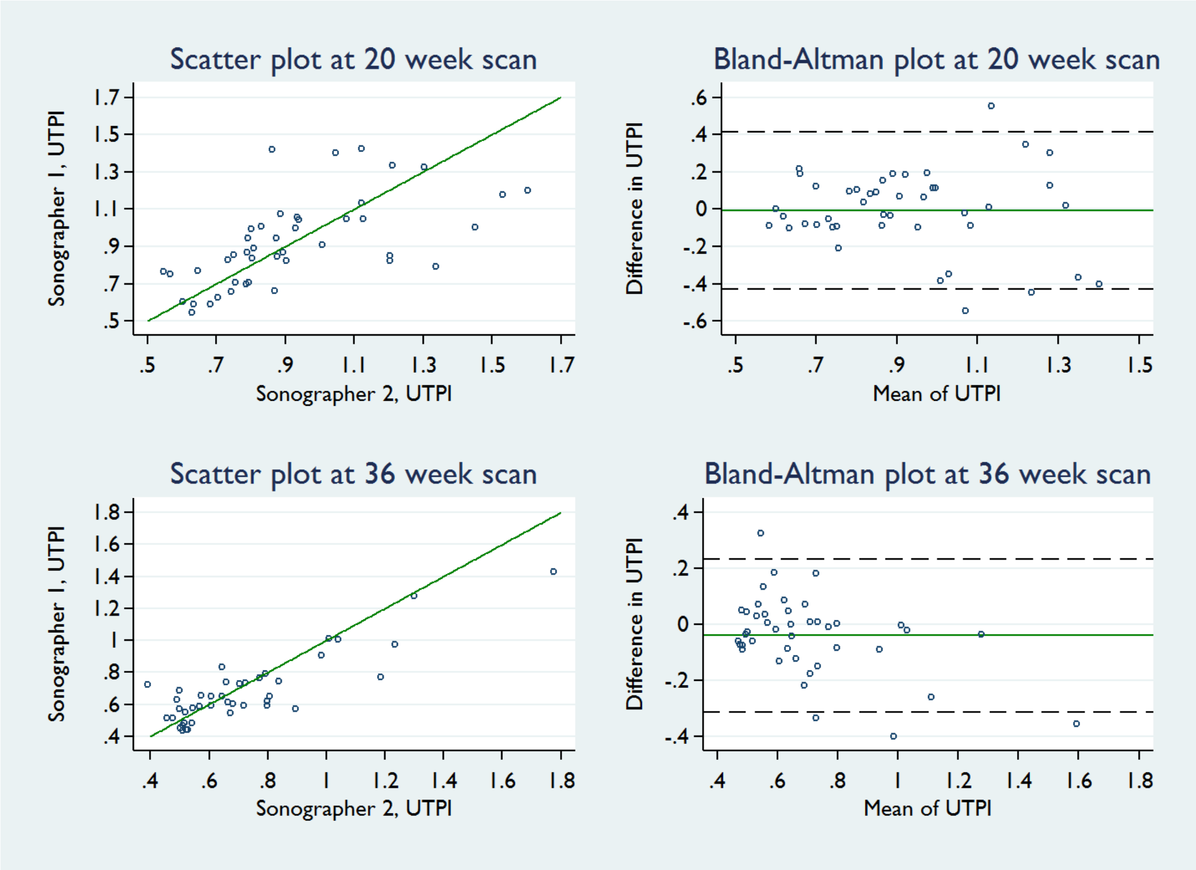

For the concordance correlation coefficient and 95% limits of agreement, refer to Supplementary Table 3.

Supplementary Figure 14. Scatter plot and Bland-Altman plot describing inter-observer repeatability in a sub-sample: umbilical artery pulsatility index (UMPI) at 20 weeks (n=45) and 36 weeks (n=43).

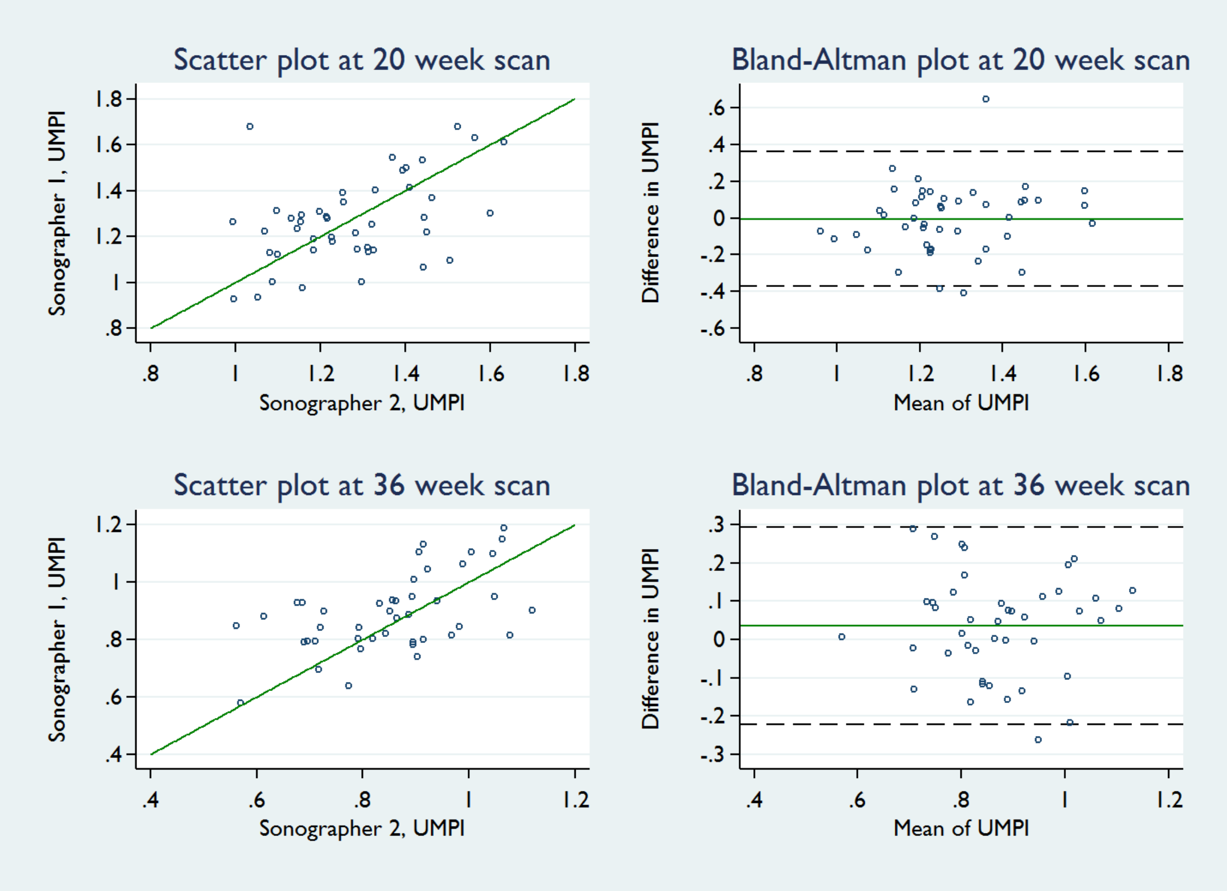

For the concordance correlation coefficient and 95% limits of agreement, refer to Supplementary Table 3.

Supplementary Figure 15. Receiver operating characteristic curve analysis of universal and selective ultrasonography.

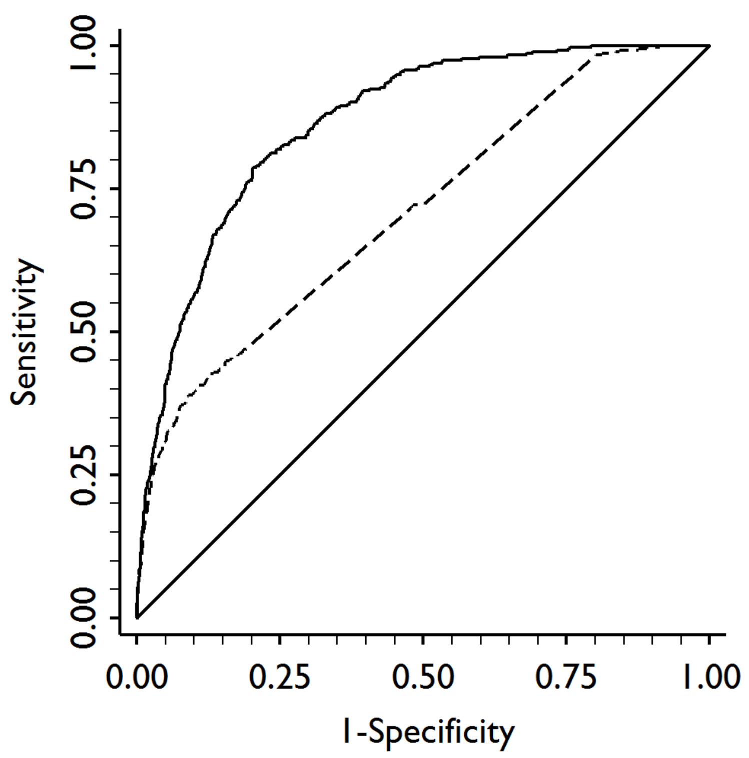

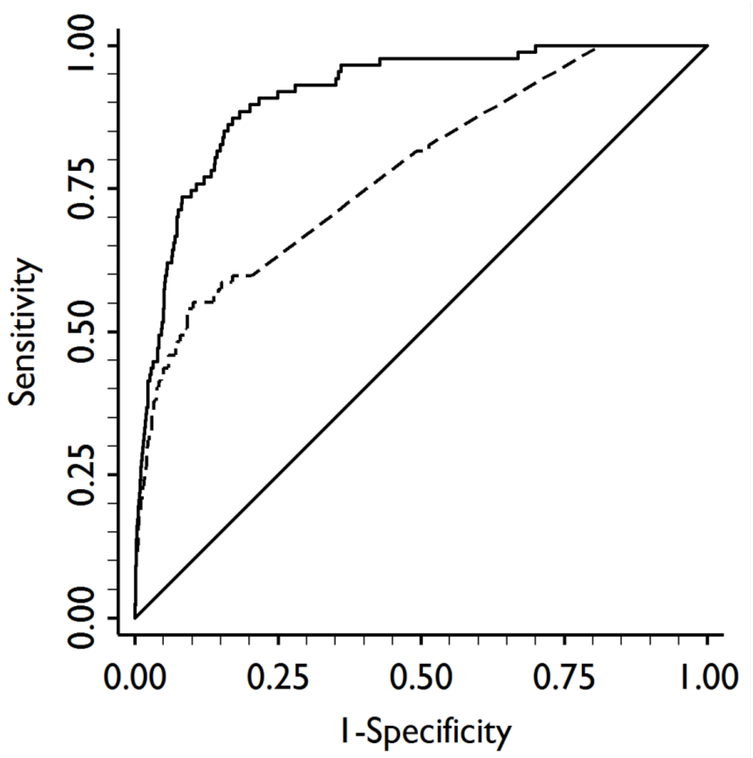

The receiver operating characteristic (ROC) curve for **A.** Small for gestational age (SGA, <10th percentile), and **B.** severe SGA (<3rd percentile), using the estimated fetal weight (EFW) percentile (i.e. the percentile from the last scan performed prior to birth). Solid lines represent universal ultrasonography and dashed lines represent selective ultrasonography. When the results of selective sonography were analysed, 58% (2,311/3,977) women did not have a clinically indicated scan at or after 26 weeks gestational age. In this group, EFW was imputed using a sex-specific population median. Areas under the ROC curves (95% confidence interval) are 0.87 (0.85-0.88) and 0.91 (0.89-0.94) for universal scan and 0.71 (0.68-0.74) and 0.78 (0.73-0.83) for selective scan, respectively. The area under the ROC curve is larger for the universal scan than for the selective scan ($p<0.0001$) for both SGA and severe SGA.

Supplementary Figure 16. Receiver operating characteristic curve analysis of universal ultrasonography, 28 week scan.

A. **B.**

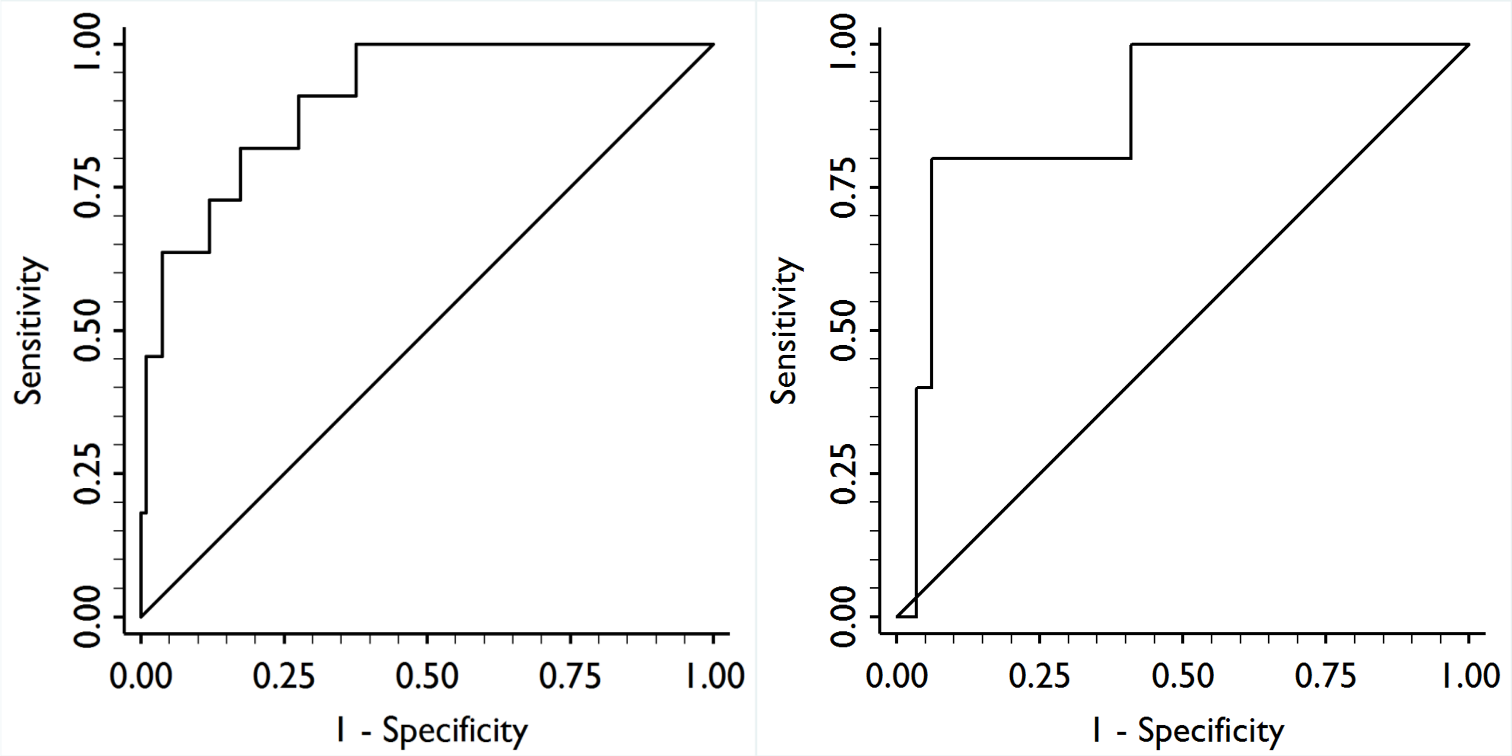

The receiver operating characteristic (ROC) curve for **A.** Small for gestational age (<10th percentile), and **B.** severe small for gestational age (<3rd percentile), using the estimated fetal weight percentile from the 28 week research scan only. The outcome in this case was delivery of an SGA or severe SGA infant prior to the 36 week research scan. Areas under the ROC curves (95% confidence interval) are 0.90 (0.82-0.99) and 0.88 (0.73-1.00), respectively.

Supplementary Figure 17. Receiver operating characteristic curve analysis of universal ultrasonography, 36 week scan.

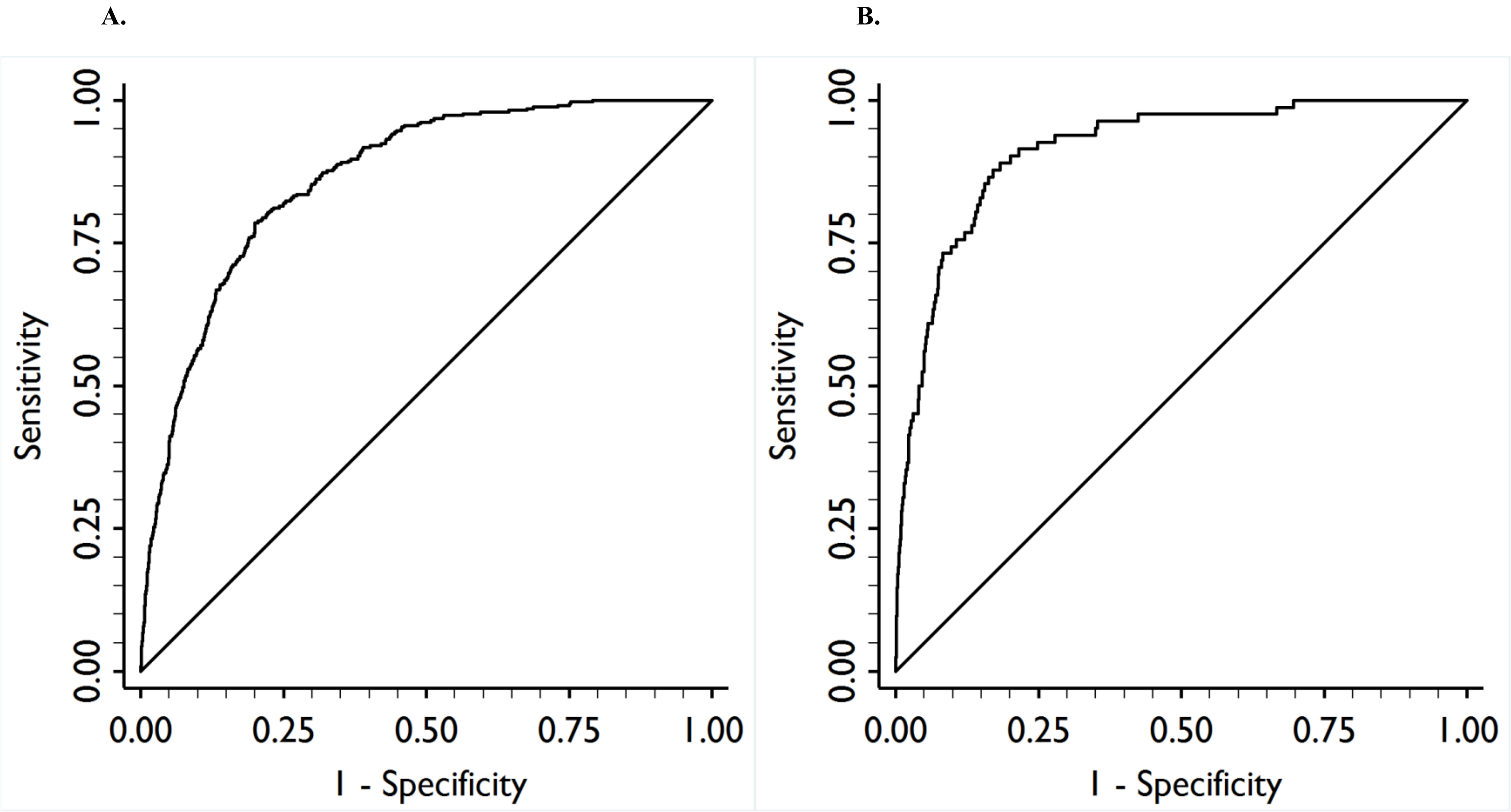

The receiver operating characteristic (ROC) curve for **A.** Small for gestational age (<10th percentile), and **B.** severe small for gestational age (<3rd percentile), using the estimated fetal weight percentile from the 36 week research scan. The outcome was, necessarily, confined to births that occurred following the 36 week research scan. Areas under the ROC curves (95% confidence interval) are 0.86 (0.85-0.88) and 0.91 (0.89-0.94), respectively.

Supplementary Table 1. Equations for mean and standard deviation (SD) estimated within the POP study (n=3,977) by gestational age interval.

Measurement	GA range	Mean model	SD model
AC	18-22 weeks	-72.60 + 11.17*GA	-5.219 + 0.5663*GA
	26-30 weeks	-44.30 + 10.09*GA	-14.40 + 0.9230*GA
	34-38 weeks	56.99 + 7.211*GA	-20.30 + 1.059*GA
BPD	18-22 weeks	-13.85 + 2.992*GA	-3.722 + 0.2822*GA
	26-30 weeks	-8.347 + 2.865*GA	4.726 - 0.06034*GA
	34-38 weeks	39.59 + 1.396*GA	-2.741 + 0.1738*GA
HC	18-22 weeks	-59.74 + 11.64*GA	-5.705 + 0.5641*GA
	26-30 weeks	2.799 + 9.393*GA	0.05039 + 0.3173*GA
	34-38 weeks	180.7 + 4.011*GA	16.01 - 0.1036*GA
FL	18-22 weeks	-24.28 + 2.782*GA	-1.994 + 0.1707*GA
	26-30 weeks	-11.29 + 2.243*GA	1.967 + 0.01064*GA
	34-38 weeks	13.93 + 1.483*GA	-3.316 + 0.1671*GA
EFW	18-22 weeks	$1459 - 172.8*GA + 5.811*GA^2$	-98.42 + 6.112*GA
	26-30 weeks	-3167 + 155.0*GA	-372.7 + 17.59*GA
	34-38 weeks	-3826 + 181.7*GA	-691.9 + 28.47*GA
AC:FL ratio	26-30 weeks	4.786 - 0.005483*GA	0.3801 - 0.004011*GA
	34-38 weeks	4.523 + 0.005143*GA	-0.6042 + 0.02453*GA
HC:AC ratio	26-30 weeks	1.331 - 0.007643*GA	0.06455 - 0.0004366*GA
	34-38 weeks	1.402 - 0.01045*GA	-0.0184 + 0.002015*GA
LN(Uterine Doppler PI)	18-22 weeks	0.7653 - 0.04311*GA	1.007 - 0.03460*GA
	26-30 weeks	-0.05423 - 0.009395*GA	0.08803 + 0.005958*GA
	34-38 weeks	0.4188 - 0.02254*GA	0.5814 - 0.008887*GA
LN(Umbilical Doppler PI)	18-22 weeks	0.9633 - 0.03650*GA	0.1636 - 0.0008369*GA
	26-30 weeks	0.8585 - 0.02892*GA	0.09365 + 0.002709*GA
	34-38 weeks	1.056 - 0.03341*GA	0.06884 + 0.003399*GA

Abbreviations: GA denotes gestational age, SD denotes standard deviation, LN denotes natural logarithm, AC denotes abdominal circumference, HC denotes head circumference, FL denotes femur length, EFW denotes estimated fetal weight and PI denotes pulsatility index.
Regression models were fitted between each measurement and GA within each GA interval (18-22, 26-30 and 34-38 completed weeks), i.e. excluding GAs without data points using published methodology.[5] Doppler PIs were log-transformed prior to model fitting. The equations of the mean and SD models give the expected mean and SD at each GA within the respective GA range. GA-specific z scores were calculated as (observed value – fitted mean) / fitted SD. Lowest and highest 10% (deciles) were determined from the z score distributions. For the AC growth velocity, change in the z score between 20 week scan and the last scan was calculated and the lowest decile of the difference was determined. For 97% of women (n=3850), the 36 week scan was the last scan. If the 36 week scan measurement was missing (delivery occurred before the 36 week scan or data were missing at the 36 week scan), the decile from the 28 week scan was used instead. Similarly for AC growth velocity, the lowest decile from the change between 20 and 28 week scans was used if the 36 week scan result was missing. For all z scores, mean=0.0 and SD=1.0. For AC growth velocity (change in z score), mean=0.0 and SD=1.1.
Z score cut-off points of the lowest decile were -1.4808 for AC velocity from 20 week scan to 36 week scan (-1.3289 from 20 week scan to 28 week scan) and -1.2732 for AC:FL ratio in the 36 week scan (-1.2638 in the 28 week scan). Z score cut-off points of the highest decile were 1.3163 for HC:AC ratio in the 36 week scan (1.2716 in the 28 week scan), 1.3231 for uterine artery Doppler PI in the 20 week scan and 1.2905 for the umbilical artery Doppler PI in the 36 week scan (1.2784 in the 28 week scan).

Supplementary Table 2. Comparisons of ultrasonographic measurements in the POP study with published reference values.

Gestational age at scan	Reference	AC (mm)	BPD (mm)	HC (mm)	FL (mm)	EFW (g)	Uterine Doppler PI	Umbilical Doppler PI
20 weeks	Published	148 (8)	47 (3)	172 (7)	31 (2)	331 (42)	1.10 (1.27)	1.27 (1.16)
	POP study	151 (6)	46 (2)	173 (6)	31 (1)	327 (24)	0.91 (1.37)	1.26 (1.16)
28 weeks	Published	233 (11)	71 (3)	260 (9)	51 (2)	1210 (154)	0.82 (1.25)	1.00 (1.20)
	POP study	238 (11)	72 (3)	266 (9)	52 (2)	1173 (120)	0.73 (1.29)	1.05 (1.18)
36 weeks	Published	313 (17)	89 (4)	319 (11)	66 (3)	2813 (357)	0.69 (1.23)	0.82 (1.23)
	POP study	317 (18)	90 (4)	325 (12)	67 (3)	2715 (333)	0.68 (1.30)	0.86 (1.21)

Abbreviations: POP denotes Pregnancy Outcome Prediction, AC denotes abdominal circumference, HC denotes head circumference, BPD denotes biparietal diameter, FL denotes femur length, EFW denotes estimated fetal weight, PI denotes pulsatility index and SD denotes standard deviation.
Data are mean (SD) for biometric measurements and geometric mean (geometric SD) for Doppler measurements. Biometric and Doppler measurements were performed as previously described.[2;6-8] AC and HC were measured using the ellipse function of the machine. BPD was measured from the outer surface (nearside to the probe) to the inner surface (far side to the probe). Umbilical Doppler was assessed in a free loop of cord in the middle of its length (i.e. outside the regions of the umbilical and placental insertions), and uterine Doppler was assessed where the vessels cross the external iliac artery and vein.
In the research scans at 28 and 36 weeks, the screen display of gestational age (GA) equivalence of measurements in the machine was disabled, to prevent ad hoc assessment of the appropriateness of growth measurements.
EFW was calculated using published formulae.[3] Where the head measurements could be made, all four parameters were employed, otherwise the equation employing AC and FL was applied.
The published reference values for AC, HC and FL are from the INTERGROWTH Project,[1] for BPD are from Chitty and Altman[2] for EFW are from Hadlock at al, 1991[4], for uterine artery Doppler are from Gomez et al, 2008[9] and for umbilical artery Doppler are from Acharya et al, 2005[10]. In the POP study, regression models were fitted within each gestational age interval to obtain means, standard deviations and percentiles (see details in Supplementary Table 1). Uterine and umbilical Doppler PIs were log-transformed prior to model fitting, hence geometric means and standard deviations are presented.

Supplementary Table 3. Analysis of reproducibility and reliability of measurements at 20 and 36 weeks gestational age.

**20 week scan**	**N**	**CCC**	**CV (%)**	**Difference: mean (95% LA)**
AC [mm]	45	0.79	1.31	-1.60 (-10.6, 7.44)
BPD [mm]	45	0.80	1.16	0.18 (-2.57, 2.93)
HC [mm]	45	0.72	1.03	2.58 (-5.90, 11.1)
FL [mm]	45	0.60	2.00	-0.16 (-3.50, 3.19)
EFW [g]	45	0.84	2.43	-3.91 (-42.8, 35.0)
Uterine Doppler PI	44	0.62	8.22	-0.007 (-0.43, 0.42)
Umbilical Doppler PI	45	0.45	5.58	-0.005 (-0.37, 0.36)
**36 week scan**	**N**	**CCC**	**CV (%)**	**Difference: mean (95% LA)**
AC [mm]	44	0.72	1.52	-5.16 (-29.4, 19.1)
BPD [mm]	36	0.95	0.46	1.19 (-1.99, 2.38)
HC [mm]	37	0.63	1.07	-2.22 (-21.8, 17.4)
FL [mm]	43	0.52	1.54	0.12 (-4.95, 5.18)
EFW [g]	43	0.77	3.15	-82.2 (-484, 319)
Uterine Doppler PI	42	0.83	7.08	-0.039 (-0.31, 0.23)
Umbilical Doppler PI	43	0.53	6.35	0.035 (-0.22, 0.29)

Abbreviations: CCC denotes Concordance Correlation Coefficient,[11] CV denotes Coefficient of Variation, BPD denotes biparietal diameter, HC denotes head circumference, AC denotes abdominal circumference, FL denotes femur length, EFW denotes estimated fetal weight calculated from BPD, HC, AC and FL measurements,[3] PI denotes Pulsatility Index and LA denotes Limits of Agreement.[12]

Summary of results

Inter-observer reliability and agreement statistics suggest a very small measurement error for fetal biometric measurements in both 20 week and 36 week scans. Differences in measurements between two sonographers were slightly larger for FL than for BPD, HC and AC. These differences had a cumulative effect on EFW which was calculated from the four measurements, resulting in a slightly higher mean coefficient of variation (CV) than was observed for individual measurements. There was more variation in Doppler measurements in both scans (mean CV range: 5.58-8.22%) than in biometric measurements (mean CV range: 0.46-3.15%). There was no clear indication that the difference in measurements between sonographers varied according to the mean of the two measurements, except for uterine artery Doppler PI measurements, where largest differences tended to occur at the top end of the distribution (Supplementary Figure 13: Bland-Altman plots).

Supplementary Table 4. Hospital record data comparing women recruited to the study with eligible women who were not recruited.

Characteristic	Recruited N=4265	Not recruited N=2909	P Value
Age, years			
<20	115 (2.7)	166 (5.7)	
20-24.9	527 (12)	550 (19)	
25-29.9	1210 (28)	876 (30)	<0.0001†
30-34.9	1637 (38)	901 (31)	
35-39.9	668 (16)	353 (12)	
≥ 40	108 (2.5)	63 (2.2)	
White ethnicity	3914 (92)	2490 (86)	<0.0001
Missing	62 (1.5)	46 (1.6)	
Smoker at booking	347 (8.1)	350 (12)	<0.0001
Missing	188 (4.4)	141 (4.9)	
BMI, kg/m^2			
<25	2339 (55)	1634 (56)	
25-29.9	1009 (24)	629 (22)	
30-34.9	330 (7.7)	198 (6.8)	0.10
35-39.9	106 (2.5)	86 (3.0)	
≥ 40	60 (1.4)	33 (1.1)	
Missing	421 (9.9)	329 (11)	
≥1 previous miscarriage (1st trimester)	540 (13)	297 (10)	0.003
≥1 previous miscarriage (2nd trimester)	108 (2.5)	57 (2.0)	0.15
Birth weight centile			
SGA (<10th)	390 (9.1)	298 (10)	0.13
Severe SGA (<3rd)	102 (2.4)	94 (3.2)	0.03
Missing	55 (1.3)	33 (1.1)	
Gestational age, weeks			
Preterm: <24	22 (0.5)	18 (0.6)	
Preterm: 24-<33	43 (1.0)	32 (1.1)	
Preterm: 33-<37	156 (3.7)	119 (4.1)	0.29†
Term: ≥ 37	4012 (94)	2727 (94)	
Missing	32 (0.8)	13 (0.4)	
Transfer to neonatal unit	216 (5.1)	176 (6.1)	0.07
Missing	34 (0.8)	18 (0.6)	
Mode of delivery			
Vaginal	2094 (49)	1537 (53)	
Assisted vaginal	1006 (24)	702 (24)	0.0002
Caesarean section	1165 (27)	670 (23)	
Outcome of birth			
Livebirth	4231 (99)	2889 (99)	
Miscarriage	5 (0.1)	3 (0.1)	
Termination of pregnancy	18 (0.4)	12 (0.4)	0.75
Stillbirth	11 (0.3)	4 (0.1)	
Missing	0 (0)	1 (0.03)	

Abbreviations: BMI denotes body mass index, SGA denotes small for gestational age.
The hospital's delivery database (PROTOS) was used to compare basic characteristics of women recruited to the study and eligible women who were not recruited.
Data are expressed as median (inter-quartile range) or n (%) as appropriate. P-values are for difference between groups calculated using the two-sample Wilcoxon rank-sum (Mann-Whitney) test for continuous variables and the Pearson Chi-square test for binary and categorical variables. †Score test for trend of odds is reported for categorical ordered variables if the trend is approximately linear. For fields where there is no category labelled "missing", data were 100% complete.

Maternal age is defined as age at delivery. Missing category is not included in statistical tests. Recruited N = 4512 minus 247 who delivered elsewhere and had no PROTOS record = 4265.
Not recruited N = 3516 eligible minus 607 without PROTOS record = 2909.

Supplementary Table 5. The area under the ROC curve (95% confidence interval) for universal ultrasonographic screening for SGA and severe SGA infants, stratified by gestational age at birth.

Research scan	GA at birth	N	SGA	Severe SGA
28 week scan	28-32 weeks	30	0.89 (0.74-1.00)	0.86 (0.73-0.99)
	33-36 weeks	133	0.92 (0.85-1.00)	0.87 (0.73-1.00)
	37-40 weeks	2558	0.77 (0.74-0.80)	0.82 (0.77-0.88)
	≥41 weeks	1254	0.76 (0.72-0.80)	0.80 (0.71-0.88)
36 week scan	37-40 weeks	2556	0.88 (0.86-0.90)	0.93 (0.90-0.96)
	≥41 weeks	1250	0.86 (0.83-0.89)	0.89 (0.83-0.94)

ROC denotes receiver operating characteristic, GA denotes gestational age and SGA denotes small for gestational age. SGA is defined as birth weight <10th percentile and severe SGA is defined as birth weight <3rd percentile. The area under the ROC curve is calculated using the estimated fetal weight percentile.

Supplementary Table 6. Sensitivity analyses for comparison of universal versus selective ultrasonography for detection of SGA (birth weight <10th percentile) infants.

	**Sensitivity analysis 1 (n=4,160)**		**Sensitivity analysis 2 (n=4,160)**		**Sensitivity analysis 3 (n=3,747)**		**Sensitivity analysis 4 (n=3,977)**	
	Selective	*Universal*	*Selective*	*Universal*	*Selective*	*Universal*	*Selective*	*Universal*
Sensitivity (%)	20	55	20	55	17	56	20	59
Specificity (%)	98	90	98	90	99	90	98	89
Positive predictive value (%)	47	35	47	35	56	36	50	35
Negative predictive value (%)	93	95	93	95	92	95	93	96
False positive rate (%)	2	10	2	10	1	10	2	11
False negative rate (%)	80	45	80	45	83	44	80	41
Positive likelihood ratio	9.3	5.6	9.3	5.6	12.9	5.7	10.3	5.5
Negative likelihood ratio	0.8	0.5	0.8	0.5	0.8	0.5	0.8	0.5

Sensitivity Analysis 1: Including women who defaulted from one or more research scans and defining their missing scan(s) as screen negative.
Sensitivity Analysis 2: Including women who defaulted from one or more research scans and, if they had a clinically indicated scan at 26-30 weeks or 34-38 weeks, the last EFW within the respective time window was used as the result of the research scan. If no clinically indicated scan was performed within that time window, the record was treated as screen negative.
Sensitivity Analysis 3: Excluding all records where the research scan result was revealed for any reason.
Sensitivity Analysis 4: Re-classifying research scan result as screen positive if the last research scan was negative but a subsequent last clinically indicated scan was screen positive.

Supplementary Table 7. Sensitivity analyses for comparison of universal versus selective ultrasonography for detection of severe SGA (birth weight <3rd percentile) infants.

	**Sensitivity analysis 1 (n=4,160)**		**Sensitivity analysis 2 (n=4,160)**		**Sensitivity analysis 3 (n=3,747)**		**Sensitivity analysis 4 (n=3,977)**	
	Selective	*Universal*	*Selective*	*Universal*	*Selective*	*Universal*	*Selective*	*Universal*
Sensitivity (%)	33	74	33	76	29	76	32	79
Specificity (%)	97	88	97	88	98	87	97	87
Positive predictive value (%)	19	12	19	12	23	12	20	12
Negative predictive value (%)	98	99	98	99	98	99	98	99
False positive rate (%)	3	12	3	12	2	13	3	13
False negative rate (%)	67	26	67	24	71	24	68	21
Positive likelihood ratio	10.8	5.9	10.8	6.1	13.2	5.9	11.4	5.9
Negative likelihood ratio	0.7	0.3	0.7	0.3	0.7	0.3	0.7	0.2

Definitions of sensitivity analyses as per Supplementary Table 6.

Supplementary Table 8. The relationship between EFW<10th percentile, abdominal circumference growth velocity (derived from an international reference standard) and perinatal outcome.

	Perinatal outcome													
	Any neonatal morbidity (n=275)		*Metabolic acidosis (n=42)*		*5 Minute Apgar <7 (n=36)*		*Neonatal unit admission (n=229)*		*SGA + any neonatal morbidity (n=49)*		*Severe adverse perinatal outcome (n=33)*		*SGA + severe adverse perinatal outcome (n=5)*	
**Research scan result**	*RR (95% CI)*	*P*	*RR (95% CI)*	*P*	*RR (95% CI)*	*P*	*RR (95% CI)*	*P*	*RR (95% CI)*	*P*	*RR (95% CI)*	*P*	*RR (95% CI)*	*P*
EFW<10th														
EFW<10th + Normal ACGV	1.2 (0.8-1.7)	0.49	0.3 (0.0-2.1)	0.25	1.1 (0.3-3.6)	0.75	1.3 (0.8-1.9)	0.27	6.4 (3.1-13.2)	<0.0001	0.4 (0.0-2.6)	0.51	9.6 (0.6-153.7)	0.18
EFW<10th + Lowest decile ACGV	2.5 (1.7-3.5)	<0.0001	3.4 (1.5-7.6)	0.007	4.5 (2.0-10.2)	0.002	2.1 (1.4-3.2)	0.002	17.6 (9.4-33.0)	<0.0001	2.5 (0.9-7.0)	0.09	33.4 (3.0-366.6)	0.009

Abbreviations: SGA denotes small for gestational age, EFW denotes estimated fetal weight, ACGV denotes abdominal circumference growth velocity, RR denotes relative risk and CI denotes confidence interval.
All EFW are based on population based percentiles.
SGA is defined as birth weight <10th percentile, screen positive is defined as EFW<10th percentile and screen negative is defined as EFW≥10th. ACGV is based on the change in the gestational age adjusted z score between the 20 week scan and the last scan before birth. Z score at each scan was calculated using growth charts generated by the Fetal Growth Longitudinal Study component of the INTERGROWTH-21st Project, an international consortium which constructed fetal growth standards using methods recommended by the WHO.[1] The lowest decile of the change in z score between the 20 week scan and the last scan was defined within the study cohort. For 97% of women (n=3850), the 36 week scan was the last scan. If the 36 week scan measurement was missing (delivery occurred before the 36 week scan or data were missing at the 36 week scan), the decile from the 28 week scan was used instead. The change in z score cut-off point of the lowest decile was -1.594 from 20 week scan to 36 week scan (-1.2255 from 20 week to 28 week scan).
Neonatal morbidity is a composite outcome, i.e. ≥1 of the three outcomes specified: metabolic acidosis (defined as pH<7.1 and a base deficit of more than 10mmol/L), 5 minute Apgar <7, neonatal unit admission. Neonatal unit admission was defined as admission to the Neonatal Intensive Care Unit, the High dependency Unit, or the Special Care Baby Unit. Severe adverse perinatal outcome is a composite outcome, i.e. ≥1 of the following outcomes specified: stillbirth (not due to congenital anomaly), neonatal death at term (not due to congenital anomaly), hypoxic ischaemic encephalopathy at term, use of inotropes at term, mechanical ventilation at term, severe metabolic acidosis at term (defined as pH<7.0 and a base deficit of more than 12mmol/L). P-values are from 2-sided Fisher's exact test.
In the analysis stratified by ACGV (defined by the INTERGROWTH-21st growth standard), an EFW<10th percentile was associated with the risk of any neonatal morbidity when the fetal ACGV was in the lowest decile (RR=4.33, 95% CI 1.96 to 9.57) but there was no association in the normal ACGV group (RR=1.12, 95% CI 0.76 to 1.66), P for interaction = 0.003.

Supplementary Table 9. The relationship between indicators of fetal growth restriction (FGR) and perinatal outcome in babies not diagnosed SGA.

	Perinatal outcome														
	Any neonatal morbidity			*Metabolic acidosis*			*5 Minute Apgar <7*			*Neonatal unit admission*			*Severe adverse perinatal outcome*		
**FGR indicator**	*n/N (%)*	*RR (95% CI)*	*P*	*n/N (%)*	*RR (95% CI)*	*P*	*n/N (%)*	*RR (95% CI)*	*P*	*n/N (%)*	*RR (95% CI)*	*P*	*n/N (%)*	*RR (95% CI)*	*P*
Umbilical Doppler															
Referent	196/3086 (6.4)	(referent)	-	29/3086 (0.9)	(referent)	-	22/3086 (0.7)	(referent)	-	167/3086 (5.4)	(referent)	-	24/3091 (0.8)	(referent)	-
Highest decile	21/326 (6.4)	1.01 (0.66-1.57)	0.91	5/326 (1.5)	1.63 (0.64-4.19)	0.37	4/326 (1.2)	1.72 (0.60-4.96)	0.31	15/326 (4.6)	0.85 (0.51-1.42)	0.61	3/327 (0.9)	1.18 (0.36-3.90)	0.74
Uterine Doppler															
Referent	191/3032 (6.3)	(referent)	-	29/3032 (1.0)	(referent)	-	25/3032 (0.8)	(referent)	-	158/3032 (5.2)	(referent)	-	21/3035 (0.7)	(referent)	-
Highest decile	22/296 (7.4)	1.18 (0.77-1.80)	0.45	4/296 (1.4)	1.41 (0.50-3.99)	0.53	0/296 (0.0)	0.00 N/A	0.16	21/296 (7.1)	1.36 (0.88-2.11)	0.18	5/298 (1.7)	2.42 (0.92-6.38)	0.08
ACGV															
Referent	207/3173 (6.5)	(referent)	-	29/3173 (0.9)	(referent)	-	24/3173 (0.8)	(referent)	-	175/3173 (5.5)	(referent)	-	24/3179 (0.8)	(referent)	-
Lowest decile	9/225 (4.0)	0.61 (0.32-1.18)	0.16	5/225 (2.2)	2.43 (0.95-6.22)	0.07	2/225 (0.9)	1.18 (0.28-4.94)	0.69	6/225 (2.7)	0.48 (0.22-1.08)	0.07	3/225 (1.3)	1.77 (0.54-5.82)	0.42
AC:FL ratio															
Referent	205/3193 (6.4)	(referent)	-	31/3193 (1.0)	(referent)	-	24/3193 (0.8)	(referent)	-	173/3193 (5.4)	(referent)	-	19/3193 (0.6)	(referent)	-
Lowest decile	12/222 (5.4)	0.84 (0.48-1.48)	0.67	3/222 (1.4)	1.39 (0.43-4.52)	0.48	2/222 (0.9)	1.20 (0.29-5.04)	0.68	9/222 (4.1)	0.75 (0.39-1.44)	0.44	2/222 (0.9)	1.51 (0.35-6.46)	0.64
HC:AC ratio															
Referent	204/3116 (6.5)	(referent)	-	32/3116 (1.0)	(referent)	-	26/3116 (0.8)	(referent)	-	170/3116 (5.5)	(referent)	-	19/3116 (0.6)	(referent)	-
Highest decile	11/232 (4.7)	0.72 (0.40-1.31)	0.33	2/232 (0.9)	0.84 (0.20-3.48)	>0.99	0/232 (0.0)	0.00 N/A	0.25	10/232 (4.3)	0.79 (0.42-1.47)	0.55	2/232 (0.9)	1.41 (0.33-6.03)	0.65

Abbreviations: FGR denotes fetal growth restriction, SGA denotes small for gestational age, AC denotes abdominal circumference, ACGV denotes abdominal circumference growth velocity, FL denotes femur length, HC denotes head circumference, RR denotes relative risk, CI denotes confidence interval and N/A denotes not applicable.
The five previously described indicators of FGR were classified as the extreme decile associated with FGR (highest or lowest, as appropriate) compared with the other 9 deciles in the cohort.
Neonatal morbidity is a composite outcome, i.e. ≥1 of the three outcomes specified: metabolic acidosis (defined as pH<7.1 and a base deficit of more than 10mmol/L), 5 minute Apgar <7, neonatal unit admission. Neonatal unit admission was defined as admission to the Neonatal Intensive Care Unit, the High dependency Unit, or the Special Care Baby Unit. Severe adverse perinatal outcome is a composite outcome, i.e. ≥1 of the following outcomes specified: stillbirth (not due to congenital anomaly), neonatal death at term (not due to congenital anomaly), hypoxic ischaemic encephalopathy at term, use of inotropes at term, mechanical ventilation at term, severe metabolic acidosis at term (defined as pH<7.0 and a base deficit of more than 12mmol/L). P-values are from 2-sided Fisher's exact test.

Supplementary Table 10. Numbers (%) of perinatal outcomes by EFW<10th percentile and abdominal circumference growth velocity (ACGV).

	**Perinatal outcome**													
	Any neonatal morbidity (n=275)		*Metabolic acidosis (n=42)*		*5 Minute Apgar <7 (n=36)*		*Neonatal unit admission (n=229)*		*SGA + Neonatal morbidity (n=49)*		*Severe adverse perinatal outcome (n=33)*		*SGA + severe adverse perinatal outcome (n=5)*	
**Research scan result**	*n/N*	*%*	*n/N*	*%*	*n/N*	*%*	*n/N*	*%*	*n/N*	*%*	*n/N*	*%*	*n/N*	*%*
EFW≥10th	217/3415	6.4	34/3415	1.0	26/3415	0.8	182/3415	5.3	18/3415	0.5	27/3421	0.8	1/3421	0.0
EFW<10th														
Population	58/562	10.3	8/562	1.4	10/562	1.8	47/562	8.4	31/562	5.5	6/563	1.1	4/563	0.7
Customized*	45/410	11.0	6/410	1.5	6/410	1.5	36/410	8.8	26/410	6.3	6/411	1.5	4/411	1.0
EFW<10th + Normal ACGV	31/388	8.0	1/388	0.3	4/388	1.0	28/388	7.2	15/388	3.9	2/389	0.5	2/389	0.5
EFW<10th + Lowest decile ACGV	27/172	15.7	7/172	4.1	6/172	3.5	19/172	11.0	16/172	9.3	4/172	2.3	2/172	1.2

Abbreviations: SGA denotes small for gestational age, EFW denotes estimated fetal weight, ACGV denotes abdominal circumference growth velocity, RR denotes relative risk and CI denotes confidence interval.
All EFW are based on population based percentiles, unless stated otherwise. *Customized percentiles of EFW were calculated using the Gestation Related Optimal Weight (GROW) Customized Weight Centile Calculator (Gardosi J & Francis A. Customized Weight Centile Calculator. GROW v 6.7 (UK), 2013 Gestation Network, www.gestation.net).
SGA is defined as birth weight <10th percentile, screen positive is defined as EFW<10th percentile, screen negative is defined as EFW≥10th, ACGV is based on the change in the gestational age adjusted z score comparing the result at 20 weeks with the last scan before birth.
Neonatal morbidity is a composite outcome, i.e. ≥1 of the three outcomes specified: metabolic acidosis (defined as pH<7.1 and a base deficit of more than 10mmol/L), 5 minute Apgar <7, neonatal unit admission. Neonatal unit admission was defined as admission to the Neonatal Intensive Care Unit, the High dependency Unit, or the Special Care Baby Unit.
Severe adverse perinatal outcome is a composite outcome, i.e. ≥1 of the following outcomes specified: stillbirth (not due to congenital anomaly), neonatal death at term (not due to congenital anomaly), hypoxic ischaemic encephalopathy at term, use of inotropes at term, mechanical ventilation at term, severe metabolic acidosis at term (defined as pH<7.0 and a base deficit of more than 12mmol/L).

Supplementary references

(1) Papageorghiou AT, Ohuma EO, Altman DG et al. International standards for fetal growth based on serial ultrasound measurements: the Fetal Growth Longitudinal Study of the INTERGROWTH-21st Project. *Lancet* 2014;**384**:869−879.

(2) Chitty LS, Altman DG, Henderson A, Campbell S. Charts of fetal size: 2. Head measurements. *Br J Obstet Gynaecol* 1994;**101**:35−43.

(3) Hadlock FP, Harrist RB, Sharman RS, Deter RL, Park SK. Estimation of fetal weight with the use of head, body, and femur measurements--a prospective study. *Am J Obstet Gynecol* 1985;**151**:333−337.

(4) Hadlock FP, Harrist RB, Martinez-Poyer J. In utero analysis of fetal growth: a sonographic weight standard. *Radiology* 1991;**181**:129−133.

(5) Altman DG, Chitty LS. Charts of fetal size: 1. Methodology. *Br J Obstet Gynaecol* 1994;**101**:29−34.

(6) Chitty LS, Altman DG, Henderson A, Campbell S. Charts of fetal size: 3. Abdominal measurements. *Br J Obstet Gynaecol* 1994;**101**:125−131.

(7) Chitty LS, Altman DG, Henderson A, Campbell S. Charts of fetal size: 4. Femur length. *Br J Obstet Gynaecol* 1994;**101**:132−135.

(8) Bhide A, Acharya G, Bilardo CM et al. ISUOG practice guidelines: use of Doppler ultrasonography in obstetrics. *Ultrasound Obstet Gynecol* 2013;**41**:233−239.

(9) Gomez O, Figueras F, Fernandez S et al. Reference ranges for uterine artery mean pulsatility index at 11-41 weeks of gestation. *Ultrasound Obstet Gynecol* 2008;**32**:128−132.

(10) Acharya G, Wilsgaard T, Berntsen GK, Maltau JM, Kiserud T. Reference ranges for serial measurements of umbilical artery Doppler indices in the second half of pregnancy. *Am J Obstet Gynecol* 2005;**192**:937−944.

(11) Lin LI. A concordance correlation coefficient to evaluate reproducibility. *Biometrics* 1989;**45**:255−268.

(12) Bland JM, Altman DG. Statistical methods for assessing agreement between two methods of clinical measurement. *Lancet* 1986;**1**:307−310.
